# Pituitary Apoplexy in Patients with Pituitary Neuroendocrine Tumors (PitNET)

**DOI:** 10.3390/biomedicines11030680

**Published:** 2023-02-23

**Authors:** Ana-Maria Gheorghe, Alexandra Ioana Trandafir, Nina Ionovici, Mara Carsote, Claudiu Nistor, Florina Ligia Popa, Mihaela Stanciu

**Affiliations:** 1 Department of Endocrinology, “C.I. Parhon” National Institute of Endocrinology, 011683 Bucharest, Romania; 2 Department of Occupational Medicine, Faculty of Medicine, University of Medicine and Pharmacy of Craiova, 200349 Craiova, Romania; 3 Department of Endocrinology, “Carol Davila” University of Medicine and Pharmacy & “C.I. Parhon” National Institute of Endocrinology, 011683 Bucharest, Romania; 4 Department 4—Cardio-Thoracic Pathology, Thoracic Surgery II Discipline, Faculty of Medicine, “Carol Davila” University of Medicine and Pharmacy & Thoracic Surgery Department, “Carol Davila” Central Emergency University Military Hospital, 013058 Bucharest, Romania; 5 Department of Physical Medicine and Rehabilitation, Faculty of Medicine, “Lucian Blaga” University of Sibiu, 550024 Sibiu, Romania; 6 Department of Endocrinology, Faculty of Medicine, “Lucian Blaga” University of Sibiu, 550169 Sibiu, Romania

**Keywords:** pituitary neuroendocrine tumors, neurosurgery, pituitary apoplexy, hormone, surgery, anticoagulant, computed tomography, headache

## Abstract

Various complications of pituitary neuroendocrine tumors (PitNET) are reported, and an intratumor hemorrhage or infarct underlying pituitary apoplexy (PA) represents an uncommon, yet potentially life-threatening, feature, and thus early recognition and prompt intervention are important. Our purpose is to overview PA from clinical presentation to management and outcome. This is a narrative review of the English-language, PubMed-based original articles from 2012 to 2022 concerning PA, with the exception of pregnancy- and COVID-19-associated PA, and non-spontaneous PA (prior specific therapy for PitNET). We identified 194 original papers including 1452 patients with PA (926 males, 525 females, and one transgender male; a male-to-female ratio of 1.76; mean age at PA diagnostic of 50.52 years, the youngest being 9, the oldest being 85). Clinical presentation included severe headache in the majority of cases (but some exceptions are registered, as well); neuro-ophthalmic panel with nausea and vomiting, meningism, and cerebral ischemia; respectively, decreased visual acuity to complete blindness in two cases; visual field defects: hemianopia, cranial nerve palsies manifesting as diplopia in the majority, followed by ptosis and ophthalmoplegia (most frequent cranial nerve affected was the oculomotor nerve, and, rarely, abducens and trochlear); proptosis (N = 2 cases). Risk factors are high blood pressure followed by diabetes mellitus as the main elements. Qualitative analysis also pointed out infections, trauma, hematologic conditions (thrombocytopenia, polycythemia), Takotsubo cardiomyopathy, and T3 thyrotoxicosis. Iatrogenic elements may be classified into three main categories: medication, diagnostic tests and techniques, and surgical procedures. The first group is dominated by anticoagulant and antiplatelet drugs; additionally, at a low level of statistical evidence, we mention androgen deprivation therapy for prostate cancer, chemotherapy, thyroxine therapy, oral contraceptives, and phosphodiesterase 5 inhibitors. The second category includes a dexamethasone suppression test, clomiphene use, combined endocrine stimulation tests, and a regadenoson myocardial perfusion scan. The third category involves major surgery, laparoscopic surgery, coronary artery bypass surgery, mitral valvuloplasty, endonasal surgery, and lumbar fusion surgery in a prone position. PA in PitNETs still represents a challenging condition requiring a multidisciplinary team from first presentation to short- and long-term management. Controversies involve the specific panel of risk factors and adequate protocols with concern to neurosurgical decisions and their timing versus conservative approach. The present decade-based analysis, to our knowledge the largest so far on published cases, confirms a lack of unanimous approach and criteria of intervention, a large panel of circumstantial events, and potential triggers with different levels of statistical significance, in addition to a heterogeneous clinical picture (if any, as seen in subacute PA) and a spectrum of evolution that varies from spontaneous remission and control of PitNET-associated hormonal excess to exitus. Awareness is mandatory. A total of 25 cohorts have been published so far with more than 10 PA cases/studies, whereas the largest cohorts enrolled around 100 patients. Further studies are necessary.

## 1. Introduction

Various complications have been reported in the relationships of pituitary neuroendocrine tumors (PitNET); an intratumor hemorrhage or infarct/infarction, also named pituitary apoplexy (PA), is a less common feature, yet with a life-threatening potential, thus its importance in early recognition and prompt intervention. Of historical note, hemorrhages associated with pituitary tumors were first described in 1898 by Pearce Bailey, and in 1950 the term “pituitary apoplexy” was introduced by Brougham et al. [1]. PA is a medical emergency presenting as sudden onset headache, loss of vision, ophthalmoplegia, and altered consciousness in relationship with a prior known or unknown pituitary mass, mostly PitNETs (usually a nonfunctioning adenoma, but also, hormonally active tumors with or without previous specific therapy) [1,2,3]. 

The incidence of PA in the general population varies from 0.2% to 0.6%, and from 2% to 12% in selected subgroups diagnosed with different types of PitNETs [4,5]. Males are affected more frequently than females; even though any age may be involved, most cases are reported within the fifth or sixth decade of life, whereas pediatric incidence remains very low [6,7].

The underlying mechanisms of PA are not completely understood yet. The classical hypothesis states that a rapidly growing tumor exceeds vascular supply [8]. Other potential contributors are vascular endothelial growth factor (VEGF), tumor necrosis factor-α (TNF-α), pituitary tumor-transforming gene (PTTG), matrix metalloproteinase-2/9 (MMP-2/9) or MMP-9, hypoxia-inducing factor (HIF-1α), a high proliferating index Ki-6 [9,10,11]; and high-mobility group box 1 (HMGB1), a nuclear DNA-binding protein with pro-inflammatory effects [12].

In terms of clinical presentations, sudden headache, often with retro-orbital location, is one of the most common presenting symptoms of PA, with an incidence of 92% up to 100% of the patients [13]. The PA-associated mechanism includes traction of intracranial pain-sensitive structures such as the dura mater, cranial nerves, blood vessels, and meningeal irritation caused by blood and necrotic tissue [14]. Other frequent symptoms are ophthalmological complaints such as visual loss, diplopia, and ophthalmoplegia [15]. Specific hormonal anomalies, caused either by hormonally active PitNETs or by PA-induced or tumor-related hypopituitarism, may also be identified at the moment of PA diagnostic [16]. Arterial hypertension, diabetes mellitus, non-endocrine major surgical procedures, head trauma, infections, and certain drugs targeting the endothelium or clotting profile are PA-associated risk factors [16]. Hemorrhage in prolactinomas is another common scenario, especially under specific medical therapy [17,18]. Cavernous sinus invasion or the presence of a large macroadenoma may also increase the risk of developing PA [18,19]. Socioeconomic factors potentially play a role in the development of PA, yet, not unanimously accepted [20].

The imaging tests used as a diagnostic aid are mostly represented by computed tomography (CT) and magnetic resonance imaging (MRI) [21]. Even though CT is used more often, MRI has a higher sensitivity [22]. The most commonly described MRI signs of PA are “sinus mucosal thickening” and “pituitary ring sign” [23]. Of note, the diagnostic of PA needs to be confirmed by imaging findings and/or associated post-operatory pathological profile after suspicion of PA is raised from a clinical presentation with regard to a neuroendocrine and/or neurologic point of view [21,22,23].

PA management involves a multidisciplinary team, as it is considered a neuroendocrine emergency. The main source of mortality is caused by acute adrenal insufficiency. Therefore, prompt glucocorticoid replacement is crucial [24]. However, the exact protocol still represents a subject of controversy. Early surgery is necessary for patients with severe visual loss since decompression may lead to a better recovery of visual dysfunction [25], but not necessarily to complete hypopituitarism recovery [26]. Other data did not identify any differences regarding the outcome of eye profile and hormonal imbalance when comparing surgical to conservative management [27,28].

### Aim

Our purpose is to overview PA as a complication of a pituitary tumor, particularly PitNETs, from clinical presentation to management and outcome.

## 2. Method

This is a narrative review of the English-language medical literature. Inclusion criteria are the following: clinically relevant papers concerning a PubMed-based search using the keywords “apoplexy”, “hemorrhage”, or “infarction” in association with “pituitary” (alternatively “hyphophyseal”); the timeline of publication is between 2012 and 2022; we included original research, either studies or case reports/series. Exclusion criteria were PA associated with the following circumstances: pregnancy and postpartum period, infection with coronavirus amid the recent COVID-19 pandemic, and non-spontaneous PA meaning PA associated with treatment for a prior known PitNET: neurosurgery, pituitary radiotherapy, and medication for hormonally active PitNETs, for instance, dopamine analogs (cabergoline, bromocriptine) for prolactinomas and somatotropinomas, respectively, somatostatin analogs (octreotide, lanreotide, and pasireotide), and growth hormone (GH) receptor antagonist pegvisomant for somatotroph PitNETs.

## 3. PitNET Complicated with PA

**Figure 1:**
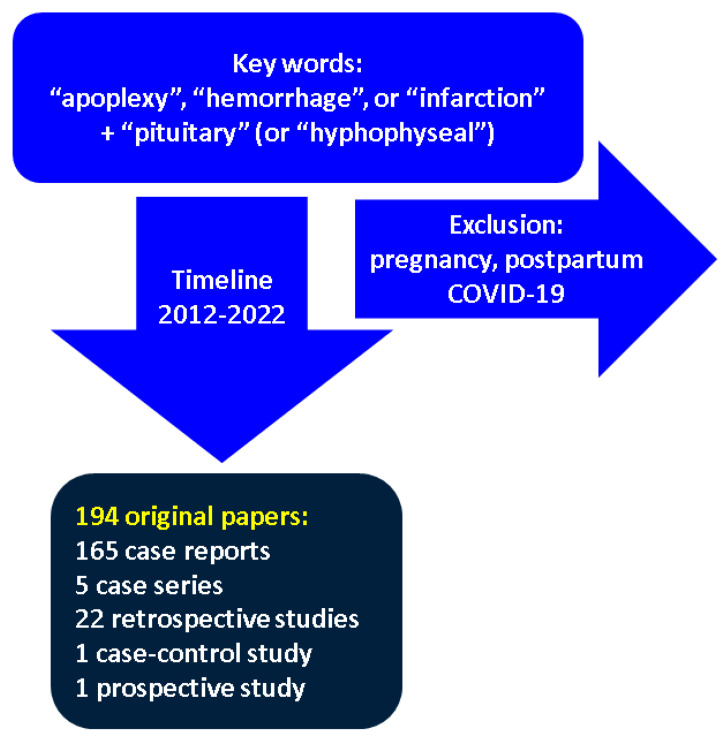
Flowchart according to our methodology.

According to our methodology, we identified 194 original papers that included 165 case reports, 5 case series, 22 retrospective studies, one case–control study, and one prospective study. Overall, 1452 patients with PA were included (926 males, 525 females, and one transgender male). In accordance with medical literature, we found a male predominance (male-to-female ratio of 1.76). The mean age of patients was 50.52 years, the youngest was 9, and the oldest was 85 years [29,30,31,32,33,34,35,36,37,38,39,40,41,42,43,44,45,46,47,48,49,50,51,52,53,54,55,56,57,58,59,60,61,62,63,64,65,66,67,68,69,70,71,72,73,74,75,76,77,78,79,80,81,82,83,84,85,86,87,88,89,90,91,92,93,94,95,96,97,98,99,100,101,102,103,104,105,106,107,108,109,110,111,112,113,114,115,116,117,118,119,120,121,122,123,124,125,126,127,128,129,130,131,132,133,134,135,136,137,138,139,140,141,142,143,144,145,146,147,148,149,150,151,152,153,154,155,156,157,158,159,160,161,162,163,164,165,166,167,168,169,170,171,172,173,174,175,176,177,178,179,180,181,182,183,184,185,186,187,188,189,190,191,192,193,194,195,196,197,198,199,200,201,202,203,204,205,206,207,208,209,210,211,212,213,214,215,216,217,218,219,220,221,222] (please see Table 1) (Figure 1).

### 3.1. Clinical Presentation in Cases with PA: Neurologic and Ophthalmic Elements

Sudden headache was the most frequent symptom and occurred in more than two-thirds of patients. (Table 1) Headache was severe, often described as the most painful headache episode an individual has ever experienced, and graded, for example, as high as 9 on a scale from 0 to 10, with 10 being the most severe in one study [31]. For practical purposes we point out that headache was absent in some cases; thus, an index of suspicion should be provided by other endocrine and non-endocrine clinical elements [43,86,196,197,198,199,200,201,202,203,204,205,206,207,208,209,210,211,212,213,214,215,216]. For instance, Enatsu et al. reported a 65-year-old woman admitted for a nonfunctioning pituitary adenoma-associated PA who presented third cranial nerve palsy and sudden decrease in visual acuity unaccompanied by headache [196]. Another example is a 65-year-old male who developed PA as a post-operative complication after a coronary artery surgery; he only presented diplopia and third cranial nerve palsy [214]. Other circumstances without headaches involve a comatose status. The case presented by Bhogal et al. involves a 63-year-old man with bradycardia and hypotension due to myxedema coma as part PA-associate hormonal picture [215]. Elsehety et al. reported a 60-year-old woman with vision loss and progression to coma due to an acute stroke as a surrounding event to PA [216].

Other common symptoms include nausea and vomiting, either with a neurological or endocrine component (acute secondary adrenal insufficiency) [25,29,30,33,36,37,38,39,40,41,42,43,44,45,46,47,48,49,50,51,52,53,54,55,56,57,58,59,60,61,62,63,64,65,66,67,68,69,70,71,72,73,74,75,76,77,78,79,80,81,82,83,84,85,86,87,88,89,90,91,92,93,94,95,96,97,98,99,100,101,102,103,104,105,106,107,108,109,110,111,112,113,114,115,116,117,118,119,120,121,128,129,130,131,132,133,134,135,136,137,138,139,140,141,142,143,144,145,146,147,148,149,150,151,152,153,154,155,156,157,158,159,160,161,162,163,164,165,166,167,168,169,170,171,174,176,178,179,180,181,182,183,184,185,186,187,188,189,190,191,192,193,194,195,196,197,198,199,200,201,202,203,204,205,206,207,208,217]. As mentioned, altered consciousness of various degrees up to coma was identified in 24 papers [56,64,70,79,93,97,100,103,111,112,114,122,123,141,151,169,174,177,189,209,210,215,216,218]. Weakness as a distinct clinical element was specified in five clinical cases (as a neurologic or endocrine consequence) [73,147,188,202,217].

Another important clinical finding is meningism, a crucial clue for differentiating between two life-threatening conditions: PA presenting as aseptic meningitis and bacterial meningitis. PA mimics neurological conditions, such as bacterial [30,37,39,59,63,78,83,106,119,168] or aseptic [100,176] meningitis, making differential diagnosis difficult. Both PA and meningitis share a similar clinical presentation with nuchal rigidity [30,59,70,78,83,119,168], positive Kernig and Brudzinski signs [37,78], fever [30,59,78,83], photophobia [39,70,83], and altered states of consciousness [30,83,106,176]. Moreover, laboratory findings consistent with bacterial meningitis, such as neutrophilic pleocytosis [30,39,59,78,119,176], and high protein content [39,59,83,119,176] were also observed in PA. In terms of glucose content of cerebrospinal fluid (CSF), patients presented with normal [59,78,83,119] or high [176] levels in CSF. In spite of findings suggestive of bacterial meningitis, all CSF cultures were sterile [30,39,59,78,83,100,119,176]. In one case, differential diagnosis was particularly difficult [78]. Villar-Taibo et al. presented a patient with clinical presentation (nuchal rigidity, positive Kernig and Brudzinski signs, and fever) and CSF analyses consistent with bacterial meningitis, whose state improved under antibiotics. Following this event, the subject was diagnosed with PA based on MRI findings and pathological examination. The authors hypothesized that meningitis might have led to PA due to vasculitis. However, it remained unclear whether PA mimicked meningitis or if previous meningitis led to PA [78]. Considering that both PA and bacterial meningitis can be life-threatening, prompt differential diagnosis and treatment are essential. Current findings suggest that the presentation of a clinical picture suggestive of meningitis includes differential diagnosis with PA, especially in patients with sterile CSF, knowing that PA may cause aseptic, irritant chemical, bacterial-like meningitis [39].

PA may associate cerebral ischemia through either extrinsic compression or vasospasm, leading to neurological deterioration (mental status changes, motor deficits, speech impairment) in patients who suffered from acute ischemic stroke following PA [79,107,146,199].

PA-associated visual disturbances include decreased visual acuity in most cases [11,25,31,32,33,37,57,70,75,80,93,96,97,98,101,102,105,110,113,120,121,128,135,146,168,177,187,190,196]. Complete blindness was reported in two papers [199,212]. Visual field defects varied from (mostly common) hemianopia [33,39,55,80,126,135,179,187,197] to cranial nerve palsies manifesting as diplopia in the majority of these cases [32,34,35,49,50,58,63,66,68,75,84,85,91,92,114,115,117,127,130,142,147,150,157,163,164,186,191,194,195,207,214] followed by ptosis [34,35,50,59,75,77,79,92,107,146,157,191,199,206] and ophthalmoplegia [47,52,95,140,158,172,190,209]. The most frequent cranial nerve affected was the oculomotor nerve, but rarely, patients also presented with abducens [25,44,77,81,84,85,91,98,113,120,121,123,124,128,142,168,174,182,192,207] and trochlear [25,63,81,84,102,121,123,124,149,168,182,209] nerves palsies. Optic chiasm compression was specified in 18 articles [32,33,46,55,62,63,65,69,80,123,125,169,177,185,186,191,192,218] or, rarely, an invasion of the cavernous sinus [44,63,65,196]. Interestingly, two patients, a 45-year-old male [34] and a 71-year-old female [207] were admitted for PA-associated proptosis.

### 3.2. PA and Hormonal Imbalance at First Diagnostic

Endocrine features in subjects admitted for PA include PitNETs-associated hormonal excess, even though most patients were nonfunctioning tumors, respectively, tumor- or PA-caused hypopituitarism (as mentioned, we did not include patients with previously recognized and treated PitNETs). In terms of clinical presentation, Cushingoid features (corticotroph PitNET) are reported in 5 studies (less than 0.005% of the patients): two males of 30, respective 33 years [31,186], a cases series of 4 females aged between 26 and 45 years [33], and other two women of 35, respective 47 years [86,161]. Acromegaly (caused by somatotroph or lactosomatotroph PitNET) was recognized in 8 patients (0.005%) aged between 26 and 50 years; a male [86,109,136,148,156,217] to female [38,78] ratio of 6 to 2. One case of gigantism (+5 SD) was reported on a 9-year-old boy with lactosomatotroph PitNET [32]. Hypopituitarism included elements of hypogonadism [127,128,129,131,135] like disturbances of menstrual cycle in women of reproductive age [171,172], etc., but, also, with a more severe potential, hypotension [29,48,83,199,210,213].

Other signs and symptoms at PA presentation include lactotroph PitNET-associated galactorrhea [141,174,183]; hyperthermia [180], pruritic skin rash associated with central adrenal insufficiency-related cortisol deficit [185], hiccups [109], epistaxis [91], hematuria [189], phonophobia [78], visual illusions [45], and symptomatic diabetic ketoacidosis [53,171,172].

In terms of the PitNET stain profile (regardless clinical expression) non-functional type was followed by somatotroph PitNETs [32,38,42,46,53,56,66,76,78,86,92,93,98,100,108,109,121,128,136,141,148,152,156,166,168,169,171,175,192,217], lactotroph PitNETs [66,75,77,90,98,100,113,121,126,128,141,166,168,169,174,175,183,202,206], gonadotroph PitNETs [71,72,77,88,91,98,99,101,105,113,114,128,179,185,187,203,221], corticotroph PitNET [31,33,92,98,100,103,118,121,128,137,144,154,155,168,174,184,186,208,220], lactosomatotroph [32,100,109], and thyrotroph PitNETs [61,92,100,105]. Other pathologic features of pituitary masses complicated with PA include: Crooke cell adenoma [130,161], tumor with switching phenotypes [143], malignant spindle and round-cell tumor [91], Rathke’s cyst [70,194], primitive neuroectodermal tumor [70], craniopharyngioma [70], and pituitary metastasis from squamous cell carcinoma [35], melanoma [55], lung and bronchogenic carcinoma [70,73], respectively, and breast carcinoma [62,187].

### 3.3. Potential Triggers and Circumstantial Events of PA

Our decade-based analysis showed that high blood pressure may be regarded as the most frequent co-morbidity (or risk factor) followed by diabetes mellitus in individuals admitted for PA. (Table 1) Two cases reported severe, complicated diabetes with diabetic ketoacidosis [53,171,172], and chronic kidney failure [59]. Another endocrine contributor was T3 thyrotoxicosis in one patient [71].

Infectious triggers were also found and include pituitary fungal infection [144], dengue hemorrhagic fever [42,74,77,103,139], varicella [208], and leptospirosis [163]. Of note, Catarino et al. presented the case of a 55-year-old woman who suffered from a corticotroph PitNET-associated PA in relationship to a fungal infection that was surgically treated without anti-fungal treatment [144]. Dengue hemorrhagic fever led to PA in five patients. The underlying mechanism is thrombocytopenia which favors bleeding at the level of PitNET. All patients had pituitary adenomas, with one corticotroph PitNET [103], one somatotroph PitNET [42], and one lactotroph–gonadotroph PitNET [77]. Three patients underwent transsphenoidal surgery (TSS) [42,74,103], one patient was treated conservatively [139], and another patient underwent TSS after initial conservative treatment with cabergoline and dexamethasone due to visual deterioration [77]. The 56-year-old patient introduced by Gohil et al. presented leptospirosis; the proposed mechanisms through which leptospirosis might induce PA include non-inflammatory vasculopathy, as well as platelet dysfunction, rather than thrombocytopenia [163]. An interesting finding was conducted by Humphreys G et al., who analyzed in a prospective study the sphenoid sinus mucosal microbiota characteristics in 10 patients with PA. The authors observed abnormal sinus bacteria like *Enterobacteriaceae* in patients with PA [105].

Traumatic causes are identified; for instance, head trauma [30,160,204], recent rugby play without an actual head trauma [168], and bodybuilding exercises [106].

Anticoagulation [25,64,66,70,128,135,138,160,166,173,195,198] and antiplatelet therapy [90,94,97,113,141,160,166,169] are the most important iatrogenic elements. For instance, we mention rivaroxaban, enoxaparin, dabigatran etexilate, warfarin, heparin, apixaban, and aspirin. Other vascular and clotting triggers include Takotsubo cardiomyopathy [213], heparin-induced thrombocytopenia [35], thrombocytopenia of other causes [97,208], and polycythemia [108]. Medical treatments in point are further on (at the statistical level of rare case reports): androgen deprivation therapy for prostate cancer [52,88,99,157,168], systemic chemotherapy like bleomycin, etoposide, cisplatin [184], doxorubicin, cyclophosphamide [207], and thyroxine therapy [61].

Oral contraceptives may contribute to PA [54]. Kobayashi et al. presented a 33-year-old female with ischemic PA of a nonfunctioning macroadenoma following the use of oral contraceptives for 1.5 years. The patient fully recovered under conservative treatment with analgesics and hormone replacement with hydrocortisone and thyroxine. The hypercoagulable state induced by estrogens was hypothesized as an underlying mechanism of PA [54]. Additionally, a vardenafil-triggered PA was reported [140]. Uneda et al. reported a 51-year-old male with signs and symptoms of PA (severe headache and oculomotor nerve palsy) the morning after taking vardenafil for erectile dysfunction for the first time in three months. He was diagnosed with apoplexy of a pituitary adenoma based on CT and MRI findings and was surgically treated. Even though the underlying mechanisms remain unclear, possible mechanisms of PA under phosphodiesterase 5 (PDE) inhibitors are hypotension, vasodilation, and antiplatelet effects [140].

Iatrogenic components may include diagnostic tests and procedures: dexamethasone suppression test [118], clomiphene use [66], combined endocrine stimulation tests [43], regadenoson myocardial perfusion scan [153], and MRI scan [46]. Among non-pituitary-surgery-related triggers, we identified major surgery [66,128,132,168,201], laparoscopic surgery [199], coronary artery bypass surgery [34,66,214], mitral valvuloplasty [84], endonasal surgery [127], and lumbar fusion surgery in prone position [80,116]. Interestingly, Naito Y et al. reported a case of PA in a 14-year-old girl with Carney complex who underwent successful resection of a cardiac myxoma, but the day after surgery she experienced headache and visual disturbances requiring urgent surgical decompression for PA [132].

Other therapeutic procedures include the intravitreal injection of vascular endothelial growth factor inhibitor [204], autologous hematopoietic cell transplantation [152], endoscopic retrograde cholangiopancreatography [124], and radiotherapy [113,166].

Two case reports presented individuals with PA caused by exposure to high altitudes (the first case was published in 2012). Both subjects were 29-year-old males without underlying pituitary conditions who ascended slowly to altitudes of over 4500 m. The initial diagnosis was acute mountain sickness in both cases. However, due to low blood pressure and findings of adrenal insufficiency, PA was suspected, and later MRI scans confirmed PA. Neither patient displayed visual disturbances [29,48]. The management included transfer to a lower altitude (as a specific approach in this distinct type of PA) as well as glucocorticoid replacements (100 mg of hydrocortisone every eight hours, the first two days, followed by 7.5 mg of prednisolone daily due to persistent hypocortisolism [29], respective 100 mg of hydrocortisone every six hours followed by full recovery [48].

**Figure 2:**
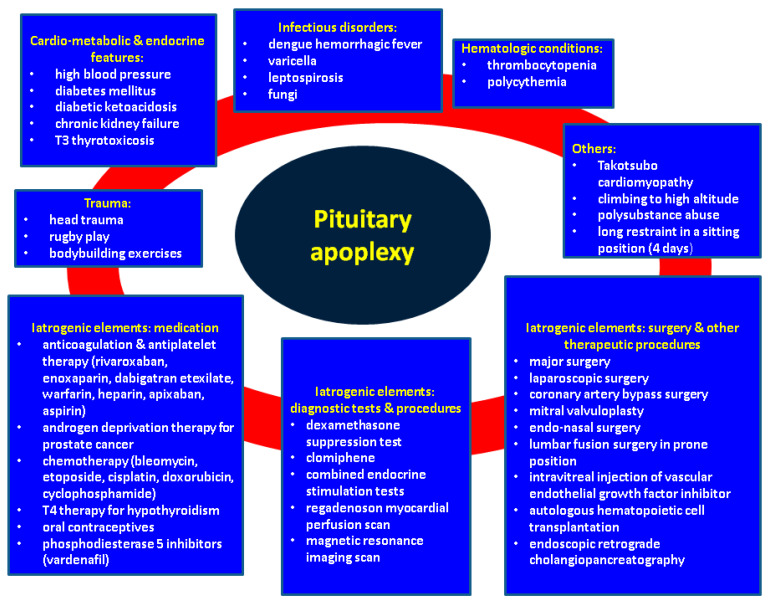
Qualitative analysis of potential contributors to spontaneous PA in PitNETs that are not previously treated (outside pregnancy and coronavirus infections). Abbreviations: T3 = triiodothyronine; T4 = thyroxine.

Of note, two unusual triggers are polysubstance abuse [191] and long restraint in a sitting position [221]. The patient presented by Sun et al., a 49-year-old male, died in custody due to a gonadotroph PitNET-associated PA following restraint in a sitting position for four days [221] (Figure 2).

### 3.4. PA Management

The management of the patients recognized with PA is summarized in Table 2. Essentially, the subjects were managed surgically (80%) or conservatively. Although the majority are TSSs, five papers introduced patients undergoing craniotomies [36,60,127,159,217]. Craniotomies were necessary for intracranial hemorrhage [36], subarachnoid hemorrhage [60], previous nasal surgery [127], and technical challenges such as cavernous sinus invasion with encasement of the internal carotid artery [159], respectively, encasing of both carotid arteries [217].

Conservative management [21,22,23,24,25,26,27,28,29,30,31,32,33,34,35,36,37,38,39,40,41,42,43,44,45,46,47,48,49,50,51,52,53,54,55,56,57,58,59,60,61,62,63,64,65,66,67,68,69,70,71,72,73,74,75,76,77,78,79,80,81,82,83,84,85,86,87,88,89,90,91,92,93,94,95,96,97,98,99,100,101,102,103,104,105,106,107,108,109,110,111,112,113,114,115,116,117,118,119,120,121,122,123,124,125,126,127,128,129,130,131,132,133,134,135,136,137,138,139,140,141,142,143,144,145,146,147,148,149,150,151,152,153,154,155,156,157,158,159,160,161,162,163,164,165,166,167,168,169,170,171,172,173,174,175,176,177,178,179,180,181,182,183,184,185,186,187,188,189,190,191,192,193,194,195,196,197,198,199,200,201,202,203,204,205,206,207,208,209,210,211,212,213,214,215,218,219,220] included vital signs monitoring, hydro-electrolytic balance, glucocorticoid substitution (intravenous hydrocortisone during the first 48 h followed by oral replacement), as well as substitution with levothyroxine and desmopressin (if needed) [123,168]. A management decision was based on the severity of clinical presentation and progression of the neurologic, eye, and hormonal features. The patients analyzed by Marx et al. were treated surgically when they presented severe visual acuity decrease, worsening of ophthalmological symptoms, resistant headache, or altered consciousness [168]. Similar criteria were applied by Almeida et al. and Bujawansa et al. [66,123]. Clinically stable individuals remained under a conservative approach [66,123,168], as well as subjects to contraindications for performing neurosurgery [123] or those refusing it [218].

A few cases under the “wait and see” approach were later referred to TSS due to worsening conditions: hematoma expansion [51] or sudden visual deterioration [77,158].

We identified three clinically relevant studies that compared conservative management versus TSS in terms of evolution [123,160,168]. Almeida et al. found that visual, cranial nerve, and endocrine outcomes are similar between the two subgroups [123]. Cavalli et al. analyzed the clinical presentation, surgical methods, and treatment outcomes of patients with PA. The authors advocated for conservative management in patients without visual impairment and suggested that a tumor with a vertical diameter greater than 35 mm should be referred to neurosurgery in the presence of visual deficits. The only statistical difference between surgically treated patients and patients under conservative treatment was the higher ratio of achieving resolution of visual field defects at the latest follow-up in the group who underwent emergency surgery [160]. Marx et al. found that following surgery, a higher number of patients presented adrenal insufficiency and hypogonadotropic hypogonadism than those who were managed conservatively, but no statistically significant difference was identified in terms of visual acuity and visual field consequences [168]. Bujawansa S et al. suggested conservative treatment as a safe and adequate approach for selected patients (those with mild and non-progressive neuro-ophthalmic defects) [66]. The findings suggest that surgical treatment wields a higher risk of associating endocrine deficiencies [168], but a higher ratio of visual field defects improvement [161], but with similar outcomes in terms of visual acuity [123,160,168].

We mention some isolated reports with uncommon management such as radiotherapy for a subject diagnosed with a null cell tumor and a gonadotroph PitNET [105]; palliative care with dexamethasone and brain radiotherapy for an individual diagnosed with bronchogenic carcinoma metastases-associated PA [73]; transfer to a lower altitude, as prior mentioned, in specific cases with climbing-induced PA [29,48]; for an intraorbital-induced PA, the patient was offered orbital surgery, preceded by embolization through direct puncture techniques combined with a transarterial approach via the right ophthalmic artery [206]. Additionally, functional PitNET continued medical therapy for specific hormonal excess with cabergoline [75,77,168,170,199,218] or somatostatin analogs [76].

### 3.5. PA-Related Outcome

A large number of patients required hormonal replacement due to PA-associated hypopituitarism manifested as hypocortisolism which is the most important aspect in terms of an immediate life-threatening approach [25,29,30,31,56,65,66,75,80,90,92,123,128,144,163,168,169,208], central hypothyroidism [25,56,65,66,72,75,101,109,123,144,163,168,169,180,184,200], and hypogonadotropic hypogonadism [25,56,65,66,72,109,128,168,169,180,198]. GH deficiency was also reported [109,128,168], whereas spontaneous acromegaly remission was reported in four papers [46,136,156,208] or partial improvement of acromegaly-related GH excess [78]. Long-term therapy with desmopressin for permanent diabetes insipidus was reported, as well as [25,31,55,66,78,90,91,111,112,113,127,128,182]. Microadenomas underlying PA had a better clinical (including endocrine) outcome when compared to macroadenomas which are also associated with a higher rate of multiple hormonal deficiencies [169]. A retrospective study by Ogawa Y et al. found that ischemic rather than hemorrhagic lesions in PA correlated with an increased progression rate [100].

As mentioned, some small studies evaluated the outcome in terms of conservative versus neurosurgical management. A retrospective study (N = 46 patients with PA) showed that individuals with non-severe neuro-ophthalmological deficits were treated conservatively (N = 27), whereas the patients with a pituitary apoplexy score (PAS) ≥4 were treated surgically (N = 19); the only statistically significant difference regarding the outcome was the higher rate of hormonal deficits in the second group [168]. Another study included 49 subjects who were referred for neurosurgery and 18 patients who were conservatively managed and showed similar improvement in visual and cranial nerve palsies [123]. A retrospective study (N = 24 subjects who underwent TSS) showed a complete tumor resection in 87.5% of cases; 94.44% of patients experienced an improvement of visual acuity; diabetes insipidus developed in 16.66% of individuals [174].

The timing of surgery was also taken into consideration by some studies, as a contributor to PA outcome. A study conducted by Rutkowski M et al. (N = 32 patients with acute PA who underwent TSS) included two groups depending on surgery timing: within 72 h of symptom onset and after 72 h; the second group had a statistically significant higher prevalence of hypopituitarism at presentation. However, in terms of hypopituitarism and visual dysfunction recovery, the outcome was similar [121].

The spontaneous resolution of PitNET through PA was reported with a favorable outcome [87,126,154,220]. Ghalaenovi H et al. published the case of a 28-year-old male with spontaneous regression of a nonfunctioning pituitary adenoma over the course of one year with the spontaneous resolution of headache, bitemporal hemianopia, and photophobia [126]. Machado et al. presented a 36-year-old female with spontaneous resolution of a corticotroph PitNET with symptoms remission within 28 months (conservative approach) [220]. Siwakoti et al. introduced a 59-year-old woman with tumor shrinkage and biochemical remission of Cushing’s disease through conservative treatment [154]. Tumor resolution with empty sella following conservative management was reported by Saberifard et al. [87].

Fatal outcome surrounding PA was registered as an early event, for instance, within 48 h [38] or after 5 years since initial TSS [91]. Overall, we identified 14 publications [35,36,38,60,62,90,91,92,100,133,182,187,216,221]. Some of the subjects were comatose at presentation [36,38,60,216], PA causing an intracerebral hemorrhage [36]; similarly, one case of the following is reported: fulminant heparin-induced thrombocytopenia [182]; fatal outcome 12 days following TSS due to sepsis [187]; or 3 months since PA in a patient with breast cancer [62] or leukemia [100]. Interestingly, two studies reported a mortality death of 1.03% (N = 97) [92], respective of 4.6% (N = 87) [90].

Of note, we identified through our analysis a single case of PA in the transgender population: a 46-year-old transgender male under testosterone therapy for 3 years who was admitted for severe headache, central hypocortisolism, and hypothyroidism. IGF-1 (insulin-like growth factor) levels, however, were high. The patient was diagnosed with somatotroph PitNET-associated PA and received surgical treatment with normalization of IGF-1 and improvement of secondary diabetes mellitus. Particularly, testosterone therapy in this situation may mask acromegaly features [76] (please see Table 2).

## 4. Discussion

### 4.1. Subentities concerning PA

An unusual case of “pneumo-apoplexy” was described by Singhal A et al.; a 65-year-old female had a PA-associated hemorrhage accompanied by pneumosella and manifested as rhinorrhea as well as classical symptoms of PA including headache and visual loss. The patient needed flap replacement of the nasoseptal defect [137].

“Recurrent” apoplexy was reported by Hosmann A et al. in 4 out of 76 patients (5.3%) after initial neurosurgery. Potential factors include residual post-operatory tumor, cavernous sinus invasion of PitNET, and ophthalmoplegia [128]. However, a recurrent tumor after initial TSS due to PA-PitNET may be found as seen in the general population with PitNET who were previously candidates for neurosurgery [145,168].

Some patients presented “subacute” PA, with little to no symptoms, thus the importance of imaging assessments like MRI scans that point out infarction or hemorrhage [166]. The term “subacute” is used for PA associated with clinical symptoms less severe than fully manifested (or “acute”) PA, thus the importance of awareness since many cases may be under-diagnosed [166,222]. Iqbal F et al. published a retrospective analysis on 55 patients (33 with acute PA and 22 with subacute PA). Severe headache and hyponatremia were more frequent in the acute group whereas the ratio of individuals who were referred to surgery was similar between the two subgroups [166]. Garg M et al. described the case of a 22-year-old female with vision reduction as the single symptom [197]. Klimko A et al. also reported a 41-year-old acromegalic male with subacute PA and panhypopituitarism [148].

### 4.2. Controversies in PA Domain

PA is an emergency that typically presents a sudden and severe headache and visual symptoms; however, subacute cases or those without headaches should not be missed. The most frequent cause is hemorrhage or ischemia in a nonfunctioning pituitary adenoma, but hormonally active PitNET may embrace a PA scenario. Middle-aged males are affected with the highest frequency, but both pediatric and elderly cases are shown in Table 1. The most common risk factors are high blood pressure, diabetes mellitus, and anticoagulation/antiplatelet therapy; however, at the level of case reports, uncommon conditions are reported as we prior pointed out. (Table 1) Differential diagnosis is sometimes crucial, as PA can mimic a number of conditions including meningitis or temporal arteritis [134]. Controversies around the exact panel of PA contributors still exist since there are still pathogenic elements that remain unclear. Whether the mentioned comorbidities as displayed in Figure 1 are directly connected to PA or are incidental is still a matter of debate.

**Figure 3:**
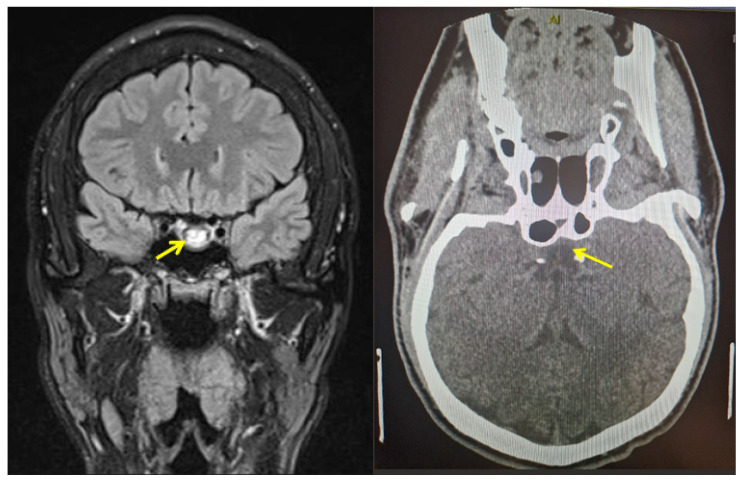
Imaging capture of a pituitary apoplexy. This is a male patient in his late 20s diagnosed with pituitary apoplexy with no prior medical or surgical history. On first presentation as an outpatient (due to severe headache), magnetic resonance imaging (performed as an emergency) shows a pituitary mass of 2.5 cm maximum diameter with inhomogeneous pattern suggesting hemorrhage (yellow arrow) at the level of a pituitary tumor (**left**). Intravenous contrast computed tomography (10 months since transsphenoidal surgery) shows a tendency to empty sella and no tumor remnants (yellow arrow) at the same level (**right**). Both captures are coronal plane.

The optimal treatment approach remains debated, but the main treatment options are TSS or conservative management. Due to possible complications from surgery, as well as a higher rate of pituitary insufficiencies after surgical treatment when compared with conservative management, careful evaluation is necessary. The individual multidisciplinary decision is mandatory. Surgical management should be reserved for patients with visual impairment due to cranial nerve palsies and optic chiasm compression, or patients with other neurological deficits. Conservative treatment is usually an adequate approach for stable patients with mild and stationary visual deficits and without further neurological impairments. When choosing conservative management, the risk of PA recurrence should also be taken into account. (Figure 3).

In terms of outcome, headaches and visual disturbances may resolve following both conservative and surgical treatment. Patients can present different degrees of anterior hypopituitarism and diabetes insipidus. Factors for a worse prognosis may be the presence of a large macroadenoma and an ischemic type of apoplexy. PA is a condition that can lead to a favorable outcome with the remission of symptoms and underlying pituitary disorder in some patients, whereas for others it can be life-threatening and invalidating when fatal cases are reported (as we already mentioned). Specific algorithms of management are still necessary.

**Figure 4:**
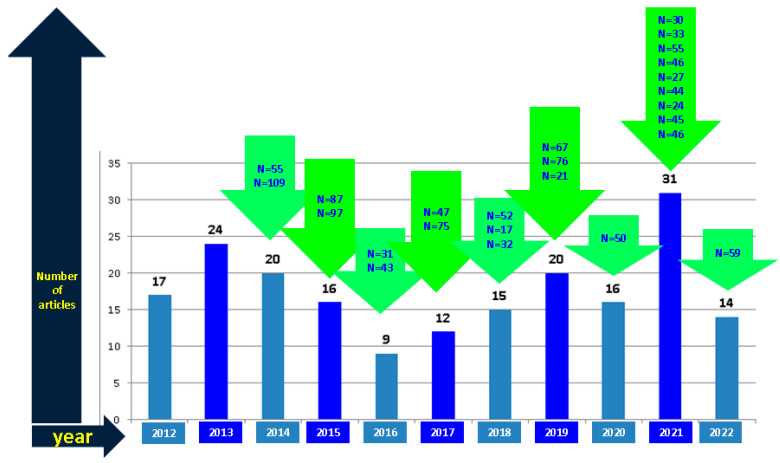
The timeline of original papers specifically addressing PA according to our methodology: the sample size analysis of original publications (please see references according to Table 1).

To our knowledge, this is the largest analysis of published cases concerning spontaneous PA (outside pregnancy and COVID-19 infection). A timeline of published papers from 2012 to 2022 according to our methodology is displayed below. The largest studies addressing individuals with PA included 109, 97, and 87 subjects, respectively [29,30,31,32,33,34,35,36,37,38,39,40,41,42,43,44,45,46,47,48,49,50,51,52,53,54,55,56,57,58,59,60,61,62,63,64,65,66,67,68,69,70,71,72,73,74,75,76,77,78,79,80,81,82,83,84,85,86,87,88,89,90,91,92,93,94,95,96,97,98,99,100,101,102,103,104,105,106,107,108,109,110,111,112,113,114,115,116,117,118,119,120,121,122,123,124,125,126,127,128,129,130,131,132,133,134,135,136,137,138,139,140,141,142,143,144,145,146,147,148,149,150,151,152,153,154,155,156,157,158,159,160,161,162,163,164,165,166,167,168,169,170,171,172,173,174,175,176,177,178,179,180,181,182,183,184,185,186,187,188,189,190,191,192,193,194,195,196,197,198,199,200,201,202,203,204,205,206,207,208,209,210,211,212,213,214,215,216,217,218,219,220,221,222]. (please see Table 1 and Table 3 and Figure 4)

Abbreviations: N represents the number of patients included in studies with at least 10 subjects with PA per article (of note: in Table 1 we used the term “case series” or “study” according to the original publication; in this figure, we strictly included the original research depending on the number of patients).

**Figure 5:**
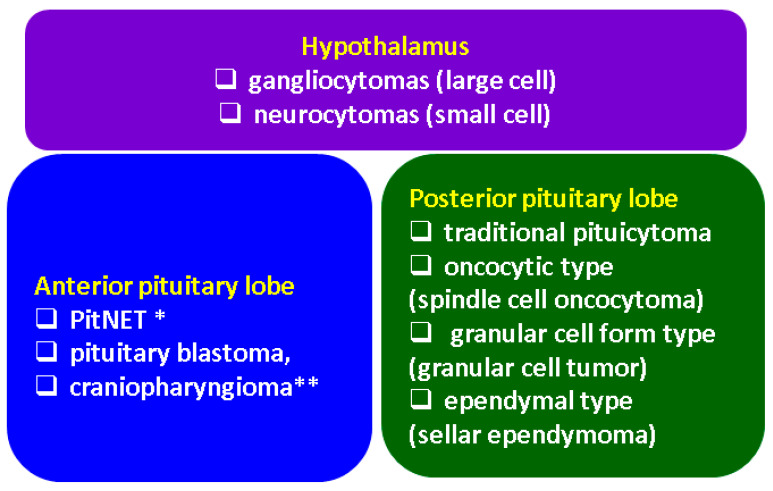
Integrating hypothalamic–pituitary tumors to new WHO 2022 criteria [223,224,225,226]. PitNET = pituitary neuroendocrine tumors; * well-differentiated adenohypophyseal tumors (formerly pituitary adenomas); ** two specific subtypes.

Notably, PitNETs terminology and associated concepts massively changed since the WHO 2022 classification. Our 10-year analysis included cases with PA in relationship with different tumors that were diagnosed according to the criteria at that time. Recently, “PitNET” replaced “pituitary adenoma” which, however, is still allowed to be used. Grossly, there are three types of tumors arising from the anterior lobe: PitNET, pituitary blastoma, and craniopharyngioma (two specific subtypes). The modern approach of these tumors massively takes into consideration the role of immunohistochemistry for mainly five elements: PIT1, TPIT, SF-1, GATA3, and ERα in order to profile PitNET subtypes. The major changes are, a part form this new terminology: null cell tumors and unclassified pluri-hormonal tumors as a subtype of PitNETs with negative staining for transcription factors; “metastatic” PitNET replaced “metastatic carcinoma” that should be differentiated from a neuroendocrine carcinoma; immature (formerly “silent”) or mature PIT1-lineage tumor are determined based on PIT1 immunostaining; and mammosomatotroph, acidophil stem cell tumors in addition to mixed somatotroph/lactotroph tumors are distinct types with respect to PIT-1 lineage of PitNET [223,224,225,226] (Figure 5).

### 4.3. Limitations

We acknowledge that the current review did not follow the PRISMA guidelines for systematic review and may have missed some studies because only PubMed was used for the literature search.

## 5. Conclusions

PA in PitNETs still represents a challenging condition requiring a multidisciplinary team from first presentation to short- and long-term management. Controversies involve the specific panel of risk factors and adequate protocols with concern to surgical decisions and their timing. The present decade-based analysis, to our knowledge the largest so far on published cases, confirms a lack of unanimous approach and criteria of intervention, a large panel of circumstantial events and potential triggers with different levels of statistical significance, in addition to a heterogeneous clinical picture (if any, as seen in subacute PA) and a spectrum of evolution that varies from spontaneous remission and control of PitNET-associated hormonal excess to exitus. Awareness is mandatory. A total of 25 cohorts have been published so far with more than 10 PA cases/studies, whereas the largest cohorts enrolled around 100 patients. Further studies are necessary.

## Figures and Tables

**Table 1:** Original studies published between 2012 and 2022 concerning PA according to our methodology; the cited studies are displayed from 2012 to 2022; the data concern the clinical presentation and potential triggers of PA in addition to underlying pituitary disease (if any) [29,30,31,32,33,34,35,36,37,38,39,40,41,42,43,44,45,46,47,48,49,50,51,52,53,54,55,56,57,58,59,60,61,62,63,64,65,66,67,68,69,70,71,72,73,74,75,76,77,78,79,80,81,82,83,84,85,86,87,88,89,90,91,92,93,94,95,96,97,98,99,100,101,102,103,104,105,106,107,108,109,110,111,112,113,114,115,116,117,118,119,120,121,122,123,124,125,126,127,128,129,130,131,132,133,134,135,136,137,138,139,140,141,142,143,144,145,146,147,148,149,150,151,152,153,154,155,156,157,158,159,160,161,162,163,164,165,166,167,168,169,170,171,172,173,174,175,176,177,178,179,180,181,182,183,184,185,186,187,188,189,190,191,192,193,194,195,196,197,198,199,200,201,202,203,204,205,206,207,208,209,210,211,212,213,214,215,216,217,218,219,220,221,222].

Author Reference Number/ Year of Publication	Type of Study	Population	Clinical Presentation at Admission for PA	Potential Triggers/Predisposing Factors	Underlying Pituitary Condition *
Brar [29] 2012	Case report	29-year-old male	Headache, nausea, non-projectile vomiting, dizziness, hypotension (systolic BP = 86–90 mmHg, diastolic BP = 56–62 mmHg)	High altitude (4572 m)	*
Cagnin [30] 2012	Case report	80-year-old male	Headache, nausea, vomiting, drowsiness, neck rigidity, fever	Head trauma hypertension	*
Chan [31] 2012	Case report	30-year-old male	Headache, decrease in visual acuity, 3rd cranial nerve palsy Clinically manifested Cushing disease		Corticotroph PitNET
Chentli [32] 2012	Case report	9-year-old boy	Retro-orbital headache, decrease in visual acuity, diplopia, papillary edema Gigantism (+5SD)		Lactosomatotroph PitNET
Choudhry [33] 2012	Case series	4 female patients (mean age: 41.75 years)	Headache (*n* = 4), nausea and vomiting (*n* = 3), decreased visual acuity (*n* = 4), bitemporal hemianopia (*n* = 4), 3rd cranial nerve palsy (*n* = 2), facial numbness/pain (*n* = 1) Clinically manifested Cushing disease (*n* = 4)	DM, hypertension	Corticotroph PitNET
Enatsu [196] 2012	Case report	65-year-old female	Left 3rd cranial nerve palsy, decrease in visual acuity		Nonfunctioning pituitary tumor
Komurcu [34] 2012	Case report	45-year-old male	Headache, bilateral 3rd cranial nerve palsy, proptosis, diplopia, loss of vision	Coronary artery bypass surgery	Null cell pituitary adenoma
Kruljac [35] 2012	Case report	77-year-old female	Headache, decrease in vision, ptosis, diplopia	Heparin-induced thrombocytopenia, 2DM, hypertension	Squamous cell carcinoma metastasis in the pituitary
Kurisu [36] 2012	Case report	68-year-old male	Headache, nausea, vomiting, 3rd cranial nerve palsy, coma	Hypertension	Nonfunctioning pituitary tumor
Liu [37] 2012	Case report	66-year-old male	Headache, vomiting, decreased visual acuity, right 3rd cranial nerve palsy, meningism		Pituitary tumor with hypopituitarism
Mohindra [38] 2012	Case report	40-year-old female	Headache, vision loss, coma Acromegalic features		Somatotroph PitNET
Paisley [39] 2012	Case report	67-year-old female	Headache, vomiting, partial loss of vision, left temporal hemianopia, light sensitivity	Hypertension	*
Tedd [40] 2012	Case report	37-year-old male	Headache, nausea, vomiting, photophobia, neck stiffness		Pituitary tumor
Verma [41] 2012	Case report	36-year-old male	Headache, fever, loss of vision, 3rd cranial nerve palsy		Pituitary tumor
Wildemberg [42] 2012	Case report	40-year-old male	Headache, vomiting	Dengue hemorrhagic fever, thrombocytopenia	Somatotroph PitNET
Yamamoto [43] 2012	Two case reports	56-year-old female	Headache, vomiting, visual disturbance, left 3rd cranial nerve palsy	Endocrine stimulation tests	Pituitary tumor
		73-year-old male	Progressive visual disturbance	Endocrine stimulation tests	Pituitary tumor
Zoli [44] 2012	Case report	59-year-old female	Headache, 6th cranial nerve palsy		Pituitary tumor
Chou [45] 2013	Case report	64-year-old female	Headache, nausea, vomiting, fever, high BP, visual illusions		Pituitary tumor
Cinar [46] 2013	Case report	38-year-old male	Headache, nausea, vomiting	DM, hypertension	Somatotroph PitNET
Delgado-Alvarado [47] 2013	Case report	70-year-old male	Headache, right total ophthalmoplegia		*
Deshwal 2013 [48]	Case report	29-year-old male	Headache, nausea, loss of appetite, fatigue, difficulty sleeping, hypotension	High altitude (5200 m)	No underlying condition
Fanous [49] 2013	Case report	39-year-old male	Headache, diplopia		Pituitary macroadenoma
Haider [50] 2013	Case report	64-year-old woman	Headache, nausea, diplopia, right ptosis	Enoxaparin	Pituitary macroadenoma
Hojo [51] 2013	Case report	29-year-old male	Headache, vomiting		Lactotroph PitNET
Huang [52] 2013	Case report	77-year-old male	Headache, nausea, vomiting, ophthalmoplegia, visual field deficit	GnRH agonist for prostate cancer	Pituitary adenoma
Jiang [53] 2013	Case report	49-year-old male	Headache	Diabetic ketoacidosis	Somatotroph PitNET
Kobayashi [54] 2013	Case report	33-year-old female	Headache, nausea, malaise	Oral contraceptives	Nonfunctioning pituitary adenoma
Machado [220] 2013	Case report	36-year-old female	Spontaneous resolution of cushingoid features under no treatment	Hypertension	Corticotroph PitNET
Masui [55] 2013	Case report	68-year-old male	Headache, fatigue, anorexia, bitemporal hemianopia		Pituitary melanoma metastasis
Mir [56] 2013	Case report	55-year-old male	Headache, vomiting, altered consciousness	2DM	Somatotroph PitNET
Mohamed [57] 2013	Case report	37-year-old male	Headache, nausea, vomiting, neck pain, 3rd cranial nerve palsy, left temporal field defect, loss of visual acuity		Pituitary adenoma
Ní Chróinín [58] 2013	Case report	75-year-old female	Headache, vomiting, diplopia, 3rd cranial nerve palsy	Hypertension, DM	Pituitary adenoma
Oh [59] 2013	Case report	42-year-old man	Headache, vomiting, fever, neck stiffness, left eye ptosis, hemianopia	Hypertension, chronic renal failure	Pituitary adenoma
Radhiana [60] 2013	Case report	44-year-old male	Headache, vomiting, coma		Pituitary adenoma
Steinberg [218] 2013	Case report	43-year-old male	Visual loss, loss of consciousness	DM, hypertension	Lactotroph PitNET
Sun [221] 2013	Case report	49-year-old male	Exitus	Long restrain in sitting position	Gonadotroph PitNET
Tutanc [61] 2013	Case report	27-year-old female	Headache, vomiting, palpitation, sleep disturbance	Thyroxine therapy	Thyrotrophic PitNET
Uemura [219] 2013	Case report	84-year-old male	Retro-orbital pain, 3rd cranial nerve palsy	DM, dabigatran etexilate	Nonfunctioning pituitary adenoma
Witczak [62] 2013	Case report	67-year-old female	Panhypopituitarism, headache, 3rd cranial nerve palsy		Pituitary metastases from breast cancer
Wong [63] 2013	Case report	62-year-old male	Headache, diplopia, 3rd, 4th, and 5th cranial nerve palsy		Pituitary adenoma
Zieliński [64] 2013	Case report	59-year-old female	Headache, vomiting, nausea, altered consciousness, visual disturbances	Anticoagulation	Nonfunctioning pituitary adenoma
Berkenstock [65] 2014	Case report	50-year-old male	Headache, nausea, vomiting, unilateral loss of vision, diarrhea, polydipsia, polyuria	Hypertension	Pituitary adenoma
Bujawansa [66] 2014	Retrospective analysis	55 patients (35 males and 20 females) Mean age: 58.4 years	Acute headache (*n* = 48) Cranial nerve palsy (*n* = 26): 3rd cranial nerve palsy (*n* = 16), 6th cranial nerve palsy (*n* = 50), multiple palsies (*n* = 5) Diplopia (*n* = 21) Visual field defect (*n* = 20) Vomiting (*n* = 14) Photophobia (*n* = 10) Nausea (*n* = 9) Facial pain/trigeminal neuralgia (*n* = 8) Collapse (*n* = 2)	hypertension (*n* = 11), anticoagulation with warfarin (*n* = 3), aspirin (*n* = 2), coronary artery bypass grafting (*n* = 2), major orthopedic surgery (*n* = 3), clomiphene (*n* = 1)	Nonfunctioning pituitary adenomas in 45 cases (82%), lactotroph PitNETs in 6 cases (11.5%), somatotroph PitNETs in 4 cases (7.2%), multiple endocrine neoplasia syndrome in 2 cases
Chao [67] 2014	Case report	14-year-old female	Headache, nausea, blurred vision		Pituitary adenoma
Cho [68] 2014	Case report	40-year-old female	Headache, diplopia, hemiparesis	Hypertension	*
Garg [197] 2014	Case report	20-year-old male	Visual impairment, bitemporal hemianopia		Pituitary adenoma
Gupta [69] 2014	Case report	62-year-old male	Headache, 3rd cranial nerve palsy, visual field defects Acute coronary syndrome, hours after admission to hospital	DM	Pituitary macroadenoma
Jho [70] 2014	Retrospective study	109 patients (69 males and 40 females) mean age: 51 years	Headache Visual acuity/field deficits Cranial nerve palsies Vomiting Altered consciousness Meningism Fever	Anticoagulation (*n* = 9)	PitNET (*n* = 98) Rathke’s cyst (*n* = 8) Primitive neuroectodermal tumor (*n* = 1) Craniopharyngioma (*n* = 1) Metastatic lung carcinoma (*n* = 1)
Lee [71] 2014	Case report	58-year-old male	Headache, visual disturbances, visual field defect, decreased sexual activity	T3 thyrotoxicosis	Gonadotroph PitNET
Maltby [72] 2014	Case report	11-year-old female	Headache, vomiting, lethargy, weight loss Tall stature, central obesity		Possible gonadotroph PitNET
Man [73] 2014	Case report	52-year-old male	Headache, left sided weakness		Bronchogenic carcinoma metastases
Mishra [74] 2014	Case report	43-year-old male	Headache, vomiting, loss of vision, bitemporal hemianopia, fever	Dengue hemorrhagic fever, thrombocytopenia	Pituitary adenoma
Mura [198] 2014	Case report	85-year-old male patient	3rd cranial nerve palsy	Laparoscopic surgery, anticoagulation (dabigatran), hypertension	Nonfunctioning pituitary adenoma
Navarro-Bonnet [75] 2014	Case report	30-year-old male	Headache, decreased visual acuity, diplopia, right ptosis Within 24 h: confusion, left hemiplegia		Giant lactotroph PitNET
Rebeiz [199] 2014	Case report	81-year-old female	Stupor, hypotension, bilateral blindness	Hypertension	Pituitary adenoma
Roerink [76] 2014	Case report	46-year-old transgender male	Headache, nausea, vomiting, blurred vision	DM during testosterone therapy	Somatotroph PitNET
Tan [77] 2014	Case report	53-year-old male	Headache, vomiting, left eye ptosis, right 6th cranial nerve palsy, right hemianopia, fever	Dengue hemorrhagic fever, thrombocytopenia	Lactotroph and gonadotroph PitNET
Villar-Taibo [78] 2014	Case report	51-year-old female	Headache, nausea, vomiting, photophobia, photophobia meningeal signs, fever	Meningitis	Somatotroph PitNET
Yoshida [200] 2014	Case report	74-year-old female	Asymptomatic apoplexy, anterior hypopituitarism, hyperthyroidism		Pituitary adenoma
Yoshino [201] 2014	Case report	78-year-old male	Fever, respiratory distress, polyuria	Thoracic surgery	Pituitary adenoma
Zhang [79] 2014	Case report	42-year-old male	Headache, fever, loss of consciousness, visual disturbance		Pituitary adenoma
Akakın [80] 2015	Case report	58-year-old male	Headache, blurred vision, bitemporal hemianopia, lethargy	Posterior lumbar fusion surgery	Pituitary adenoma
Asaithambi [81] 2015	Case report	54-year-old male	Headache, visual loss, partial 3rd, 4th, and 6th cranial nerve palsies		Pituitary adenoma
Banerjee [82] 2015	Case report	56-year-old female	Headache, visual loss, followed by neurological deterioration (unresponsive, fixed and dilated right pupil, decerebrate response to stimuli)	Fall from standing	Pituitary macroadenoma
Fountas [83] 2015	Case report	45-year-old male	Headache, fever, photophobia, hypotension, confusion		Pituitary adenoma
Kasl [202] 2015	Case report	14-year-old female	Upper limb weakness, mental status changes		Lactotroph PitNET
Kasl [203] 2015	Case report	74-year-old female	Unilateral vision loss, 3rd cranial nerve palsy	Intravitreal injection of vascular endothelial growth factor inhibitor	Gonadotroph PitNET
Kim [84] 2015	Case report	69-year-old male	Headache, visual field defect, diplopia, 3rd, 4th, and 6th cranial nerve palsy	Mitral valvuloplasty	Pituitary adenoma
Man [85] 2015	Case report	82-year-old male	Headache, diplopia, bilateral 3rd and 6th cranial nerve palsies		Pituitary adenoma
Roerink [86] 2015	Two case reports	41-year-old male	Neck pain, acromegalic features	DM	Somatotroph PitNET
		47-year-old female	Headache, a second episode of headache and visual impairment, Cushingoid features	DM, hypertension	Corticotroph PitNET
Saberifard [87] 2015	Case report	50-year-old female	Headache, vomiting, visual field defect		Pituitary adenoma
Sasagawa [88] 2015	Case report	62-year-old male	Headache, 3rd cranial nerve palsy	GnRH agonist for prostate cancer	Gonadotroph PitNET
Sasaki [89] 2015	Case report	65-year-old male	Headache, visual impairment, symptoms of adrenal insufficiency		Pituitary adenoma
Singh [90] 2015	Retrospective analysis	87 patients (55 males and 30 females) mean age: 50.9 years	Headache (*n* = 78, 89.7%) Cranial nerve palsy (*n* = 34, 39%) Visual field defects (*n* = 30, 34.1%)	Hypertension (*n* = 34, 39%) DM (*n* = 11, 12.6%) cardiothoracic surgery (*n* = 2, 2.3%) anticoagulant therapy (*n* = 9, 10.3%): heparin (*n* = 2, 22.2%) or warfarin (*n* = 7, 77.8%) antiplatelet drugs (*n* = 17, 19.5%)	Null cell (*n* = 18) Lactotroph PitNET (*n* = 8)
Teasdale [91] 2015	Case report	72-year-old male	Headache, nausea, vomiting, visual disturbances 5 years after the initial presentation: headache, vomiting, visual disturbances, diplopia, 3rd and 6th cranial nerve palsy, epistaxis		Thyreotroph, gonadotroph PitNET 5 years after the initial presentation: malignant spindle and round-cell tumor
Zhu [92] 2015	Case-control study	2021 patients with pituitary tumors, out of which: 97 cases with PA (70 males and 27 females) mean age: 50.1 ± 13.9 years (PA), randomly matched with 194 controls	Headache (*n* = 84, 86.6%) Visual deterioration (*n* = 60, 61.86%) Vomiting (*n* = 39, 40.21%) Ptosis (*n* = 25, 25.77%) Diplopia (*n* = 6, 6.18%)	Hypertension DM (differences between cases and controls were not statistically significant)	Null (non-functional) PitNET (*n* = 63) Lactotroph PitNET (*n* = 7) Somatotroph PitNET (*n* = 8) Corticotroph PitNET (*n* = 1) Thyrotroph PitNET (*n* = 2) Gonadotroph PitNET (*n* = 10) Others (*n* = 4) Multiple staining (*n* = 2)
Zou [93] 2015	Case report	23-year-old male	Headache, nausea, decreased visual acuity, loss of consciousness, fever		Somatotroph PitNET
Choudhury [94] 2016	Case report	75-year-old male	Headache, nausea, vomiting, photophobia	Antiplatelet drug	Pituitary adenoma
Doglietto [95] 2016	Case report	76-year-old female	Headache, visual disturbances, ophthalmoplegia, 3rd cranial nerve palsy	Anticoagulant therapy (dabigatran)	Nonfunctioning pituitary adenoma
Gambaracci [96] 2016	Case report	55-year-old female	Headache, decreased visual acuity, fever		Pituitary adenoma
Giammattei [97] 2016	Case series	8 male patients mean age: 70 years	Headache (*n* = 7) Nausea and vomiting (*n* = 4) Decreased visual acuity (*n* = 2) Ophthalmoplegia (*n* = 6) Altered consciousness (*n* = 1) Photophobia (*n* = 1)	Anticoagulant (*n* = 3) Antiplatelet (*n* = 1) Hypertension (*n* = 5) DM (*n* = 2) Autoimmune hemolytic anemia with thrombocytopenia (*n* = 1)	Pituitary adenoma
Giritharan [98] 2016	Case series	31 patients (19 males, 12 females), mean age: 55 years	Headache (*n* = 31, 100%) Nausea/vomiting (*n* = 17, 55%) Visual field defect (*n* = 18, 58%) Decrease in visual acuity (*n* = 7, 23%) Ocular paresis (*n* = 12, 39%): 3rd cranial nerve (*n* = 8, 26%), 6th cranial nerve (*n* = 6, 19%)	Hypertension (*n* = 5, 16%) Oral anticoagulation (*n* = 3, 10%) Heparin therapy (*n* = 1, 3%) Pregnancy (*n* = 1, 3%) Previously known adenoma (*n* = 1, 3%)	Nonfunctioning adenomas (*n* = 21, 67.74%) Somatotroph PitNET (*n* = 2, 6.45%) Lactotroph PitNET (*n* = 1, 3.22% ) Gonadotroph PitNET (*n* = 5, 16.12%) Corticotroph PitNET (*n* = 2, 6.45%)
Keane [99] 2016	Case report	67-year-old male	Headache, 3rd cranial nerve palsy	GnRH agonist for prostate cancer	Gonadotroph PitNET
Ogawa [100] 2016	Retrospective study	43 patients (30 males and 13 females) mean age: 56.67 years	Headache Cranial nerve palsies Aseptic meningitis Altered consciousness		Nonfunctioning pituitary adenoma (*n* = 29) Lactosomatotroph PitNET (*n* = 5) Lactotroph PitNET (*n* = 4) Thyrotroph PitNET (*n* = 3) Somatotroph PitNET (*n* = 1) Corticotroph PitNET (*n* = 1)
Paschou [101] 2016	Case report	37-year-old male	Headache, nausea, fever, visual acuity decrease, 3rd cranial nerve palsy, neck stiffness, confusion		Gonadotroph PitNET
Sussman [102] 2016	Case report	46-year-old male	Headache, dizziness, decrease in visual acuity, syncope, 3rd and 4th cranial nerve palsy, hemiparesis	Anti-hypertensive drugs	Pituitary adenoma
Balaparameswara Rao [103] 2017	Case report	45-year-old male	Headache, vomiting, altered consciousness	Dengue hemorrhagic fever	Corticotrophic PitNET
Grangeon [104] 2017	Case report	83-year-old male	Headache (hemicrania)	DM	Pituitary adenoma
Humphreys [105] 2017	Prospective study	10 patients, out of which 5 patients with PA (2 males and 3 females) mean age: 46 years	Headache (*n* = 3) Visual field defect (*n* = 1) Decreased visual acuity (*n* = 1) Irregular menses (*n* = 1) Hyponatremia (*n* = 1) Altered consciousness (*n* = 1)		Gonadotroph PitNET (*n* = 3) Thyrotroph PitNET (*n* = 1) Null cell (*n* = 1)
Ishigaki [204] 2017	Case report	66-year-old male	Delayed 3rd cranial nerve palsy	Head trauma, hypertension, DM	Nonfunctioning pituitary adenoma
Law-Ye [106] 2017	Case report	29-year-old male	Headache, progression to coma	Bodybuilding exercises	Pituitary adenoma
Pasha [107] 2017	Case report	35-year-old female	Headache, vomiting, 3rd cranial nerve palsy, decrease in vision, acute right side motor deficits and speech impairment		Pituitary adenoma
Patra [108] 2017	Case report	36-year-old male	Headache	Polycythemia	Somatotroph PitNET
Rais [205] 2017	Case report	86-year-old female	Functional decline	Hypertension, DM	Pituitary adenoma
Simsek Bagir [109] 2017	Case report	32-year-old male	Headache, nausea, vomiting, hiccups, acromegalic features		Lactosomatotroph PitNET
Souteiro [110] 2017	Case report	77-year-old female	Headache, nausea, vomiting, psychomotor impairment, visual acuity loss	Hypertension, 2DM	Corticotroph PitNET
Waqar [111] 2017	Retrospective study	47 patients with pituitary apoplexy (33 males and 14 females) mean age: 54 ± 15 years - patients were compared with 50 surgically treated patients with nonfunctioning pituitary adenomas	Headache (*n* = 42) Nausea/vomiting (*n* = 25) Visual field defect (*n* = 26) Visual acuity defect (*n* = 18) Cranial nerve palsy (*n* = 19) Altered consciousness (*n* = 4)	Hypertension (*n* = 11) Anticoagulation-antiplatelet therapy (*n* = 4)	Pituitary adenoma
Zoli [112] 2017	Retrospective study	75 patients (45 males and 30 females) mean age: 52.4 ± 16.2 years	Headache (*n* = 75, 100%) Anterior hypopituitarism (*n* = 51, 68%) Visual disturbances (*n* = 55, 73.4%) Ophthalmoplegia (*n* = 38, 50.7%) Altered consciousness (*n* = 2, 2.6%)		Pituitary adenoma
Abbara [113] 2018	Retrospective study	52 patients (25 males and 27 females) mean age: 46.7 years	Headache (*n* = 40/43) Vomiting (*n* = 22/43) 3rd cranial nerve palsy only (*n* = 12/35) 6th cranial nerve palsy only (*n* = 8/35) 3rd and 6th palsy (*n* = 3/35) Decreased visual acuity (*n* = 14/35) Visual fields defects (*n* = 13/35)	Hypertension (*n* = 17) Intrapartum/puerperal (*n* = 7) DM (*n* = 4) Antiplatelet or anticoagulant (*n* = 3) Dopamine agonists (*n* = 2) Radiotherapy (*n* = 2) None (*n* = 24)	Nonfunctioning adenoma or gonadotroph PitNET (*n* = 47) Lactotroph PitNET (*n* = 5)
Bettag [114] 2018	Case report	75-year-old female	Headache, diplopia, decreased consciousness		Gonadotroph PitNET
Fan [115] 2018	Case report	79-year-old male	Headache, decreased vision, diplopia, 3rd cranial nerve palsy		Pituitary adenoma
Hodgson [206] 2018	Case report	71-year-old female	Proptosis		Lactotroph PitNET
Jang [207] 2018	Case report	41-year-old female	Diplopia, visual disturbances, 6th cranial nerve palsy	Chemotherapy (doxorubicin and cyclophosphamide)	Pituitary macroadenoma
Joo [116] 2018	Case report	73-year-old male	Headache, 3rd cranial nerve palsy	Lumbar fusion surgery in prone position	Pituitary macroadenoma
Komshian [117] 2018	Case report	56-year-old male	Headache, diplopia, 3rd cranial nerve palsy		Nonfunctioning pituitary adenoma
Kuzu [118] 2018	Case report	30-year-old male	Headache, 3rd cranial nerve palsy	Dexamethasone suppression test	Corticotroph PitNET
Myla [119] 2018	Case report	59-year-old male	Headache, stiff neck, nausea	Hypertension	Nonfunctioning pituitary macroadenoma
Raj [208] 2018	Case report	18-year-old male	Vomiting, adrenal insufficiency	Varicella infection, thrombocytopenia	Corticotroph PitNET
Ricciuti [120] 2018	Case series	17 patients (12 males and 5 females) mean age: 58.76 years	Headache (*n* = 5) Vomiting (*n* = 4) 3rd cranial nerve palsy (*n* = 10) 6th cranial nerve palsy (*n* = 4) Visual acuity deficit (*n* = 4) Neck stiffness (*n* = 1)	Hypertension (*n* = 3) Previous radiation therapy (*n* = 1)	
Rutkowski [121] 2018	Retrospective study	32 patients (21 males and 11 females) mean age: 49 years	Headache (*n* = 32, 100%) Nausea/vomiting (*n* = 10, 31%) Encephalopathy (*n* = 6, 19%) Nuchal rigidity (*n* = 4, 12%) Hypopituitarism (*n* = 28, 88%) Decrease in visual acuity (*n* = 31, 97%) Cranial nerve palsy (3rd, 4th and/or 6th) (*n* = 16)		Nonfunctional adenoma (70%) Clinically hypersecreting adenoma (15%) Lactotroph PitNET Somatotroph PitNET Corticotroph PitNET
Salehi [209] 2018	Case report	78-year-old male	Ophthalmoplegia, 3rd and 4th cranial nerves palsy, altered consciousness		Pituitary adenoma
Ward [210] 2018	Case report	63-year-old male	Fever, hypotension, tachycardia, altered consciousness	Closed head injury	Nonfunctioning pituitary adenoma
Yamada [122] 2018	Case report	50-year-old male	Headache, visual impairments, loss of consciousness		*
Almeida [123] 2019	Retrospective analysis	67 patients (41 males and 26 females) mean age: 57.4 +/− 16.2 years	Headache (*n* = 60) Visual deficit (*n* = 44) Hypopituitarism (*n* = 40) Cranial nerve palsy (*n* = 32): 3rd (*n* = 17), 4th (*n* = 8), 6th (*n* = 8) Altered levels of consciousness (*n* = 10)		Pituitary adenoma
Crisman [124] 2019	Case report	43-year-old male	Headache, cranial nerve palsies (3rd, 4th, and 6th)	Endoscopic Retrograde Cholangiopancreatography	Pituitary adenoma
Dupont [125] 2019	Case report	83-year-old female	Headache, bilateral vision loss	Dual anti-aggregation	Pituitary adenoma
Ghalaenovi [126] 2019	Case report	28-year-old male	Resolution of initial symptoms (headache, nausea, photophobia, bitemporal hemianopia at diagnosis of a pituitary macroadenoma)		Lactotroph PitNET
Harju [127] 2019	Case report	48-year-old male	Headache, diplopia, 3rd cranial nerve palsy, visual field defect, fever	Endoscopic endonasal surgery	Non-functional macroadenoma
Hosmann [128] 2019	Retrospective analysis	76 patients (53 males and 23 females) mean age: 53.7 +/−14.3 years	Headache (*n* = 63, 82.9%) Nausea/vomiting (*n* = 26, 34.2%) Decrease in visual acuity (*n* = 42, 54.9%) Visual field deficit (*n* = 48, 63.3%) Cranial nerve palsy: 3rd (*n* = 35, 46.1%), 6th (*n* = 22, 28.9%) Altered levels of consciousness (*n* = 12, 15.8%)	Hypertension (*n* = 23, 30.3%) Oral anticoagulation (*n* = 14, 18.4%) DM (*n* = 9, 11.8%) Extracranial surgery within 24 h before apoplexy (*n* = 4, 5.3%)	Clinically nonfunctioning PitNETs (81%): gonadotroph PitNET (37.9%), null-cell (29.3%), plurihormonal (8.6%), corticotroph (3.5%), somatotroph (1.7%) Clinically functioning PitNETs: lactotroph (10.4%), corticotroph (6.9%), somatotroph (1.7%)
Kirigin Biloš [129] 2019	Case report	74-year-old male	Headache, nausea, vomiting, vertigo, 3rd cranial nerve palsy		Pituitary adenoma
Krug [130] 2019	Case report	45-year-old male	Headache, diplopia		Crooke cell adenoma
Mittal [131] 2019	Case report	38-year-old male	Headache, nausea, 3rd cranial nerve palsy, visual field deficits	DM	Nonfunctioning pituitary adenoma
Naito [132] 2019	Case report	14-year-old female	Headache, visual impairment	Cardiac surgery	Pituitary adenoma
Nioi [133] 2019	Case report	50-year-old female	Headache, visual impairment, vertigo Hemodynamic collapse after placement of nasogastric tube	TSS, incorrect placement of nasogastric tube	Pituitary adenoma
Pedro [134] 2019	Case report	79-year-old male	Headache, photophobia, vomiting		Pituitary adenoma
Santos [135] 2019	Case report	74-year-old female	Headache, vomiting, decrease in visual acuity, bitemporal hemianopia	Systemic anticoagulation, DM	Pituitary adenoma
Sanz-Sapera [136] 2019	Case report	50-year-old male	Headache, acromegalic features		Somatotroph PitNET
Singhal [137] 2019	Case report	65-year-old female	Headache, vision loss, rhinorrhea		Corticotroph PitNET
Swaid [138] 2019	Case report	65-year-old female	Headache, 3rd cranial nerve palsy	Coronary angiography, anticoagulation (heparin), 2DM, hypertension	Pituitary adenoma
Thomas [139] 2019	Case report	85-year-old male	Headache, 3rd cranial nerve palsy	Dengue fever-induced thrombocytopenia	Pituitary adenoma
Uneda [140] 2019	Case report	51-year-old male	Headache, 3rd cranial nerve palsies (ophthalmoplegia)	Vardenafil therapy	Pituitary adenoma
Wang [141] 2019	Case report	21 patients (15 males and 6 females), with a mean age of 50.7 ± 15.0 years	Headache (*n* = 21) Nausea (*n* = 15) Vomiting (*n* = 14) Visual disturbances: visual field defects (*n* = 17) decreased visual acuity (*n* = 17) Cranial nerves palsies (*n* = 10) Electrolyte disturbances (*n* = 12) Menstrual disturbances and galactorrhea (*n* = 4/6) Fever (*n* = 3) Altered consciousness (*n* = 3)	Hypertension (*n* = 9) Coagulation disturbances (*n* = 9) Diabetes mellitus (*n* = 4) Antiplatelet therapy (*n* = 1) Chronic renal insufficiency (*n* = 1) Atrial fibrillation (*n* = 1) Old myocardial infarction (*n* = 1)	Nonfunctioning pituitary adenoma (*n* = 18) Lactotroph PitNET (*n* = 2) Somatotroph PitNET (*n* = 1)
Waqar [142] 2019	Case report	51-year-old male	Headache, vomiting, diplopia, 6th cranial nerve palsy		Nonfunctioning pituitary adenoma
Ahn [211] 2020	Case report	78-year-old male	Stupor, hemiparesis	Hypertension	Pituitary adenoma
Brown [143] 2020	Case report	56-year-old male	2 episodes of acute-onset headache, vomiting, and a cranial nerve palsy		Switching phenotypes
Catarino [144] 2020	Case report	55-year-old female	Headache, 3rd cranial nerve palsy	DM, Pituitary fungal infection	Corticotroph PitNET
Eichberg [145] 2020	Case report	46-year-old female	Headache, nausea, vomiting, blurred vision		Nonfunctioning pituitary adenoma
Elarjani [146] 2020	Case report	31-year-old male	Headache, hemiparesis, unilateral decreased visual acuity		Pituitary adenoma
Franzese [147] 2020	Case report	60-year-old male	Headache, nausea, weakness, diplopia, 3rd cranial nerve palsy	Coronary artery bypass grafting	Null cell adenoma
Klimko [148] 2020	Case report	41-year-old male	Headache, fatigue, weight loss		Somatotroph PitNET
Lee [149] 2020	Case report	75-year-old male	Headache, vomiting, dizziness, 3rd, 4th, and 5th nerve palsies	Hypertension, DM, hemodialysis	Nonfunctioning pituitary adenoma
Marzoughi [150] 2020	Case report	70-year-old male	Headache, diplopia, 3rd cranial nerve palsy	Hypertension, DM, anticoagulant	Pituitary adenoma
Pangal [151] 2020	Retrospective study	50 patients (31 males, 19 females) mean age: 53 years	Headache (86%) Vision loss (62%) Cranial nerve palsy (40%) Decrease in consciousness (14%)		Pituitary adenoma
Patel [152] 2020	Case report	60-year-old male	Headache, nausea, vomiting, monocular vision loss	Autologous hematopoietic cell transplantation	Somatotroph PitNET
Romano [212] 2020	Case report	65-year-old male	Blindness, hemiparesis, decreased alertness		Pituitary macroadenoma
Shetty [153] 2020	Case report	49-year-old female	Headache, palpitations, nausea, vomiting, neck stiffness	Regadenoson myocardial perfusion scan	Nonfunctioning pituitary adenoma
Siwakoti [154] 2020	Case report	59-year-old female	Headache, nausea, dizziness		Corticotroph PitNET
van Boven [155] 2020	Case report	31-year-old female	Headache, nausea, vomiting	2DM	Corticotroph PitNET
Yang [213] 2020	Case report	70-year-old female	Confusion, hypotension, fever, chills, cough		Pituitary microadenoma
Alam [156] 2021	Case report	40-year-old male	Headache acromegalic feats		Somatotroph PitNET
Aljabri [157] 2021	Case report	74-year-old male	Headache and vomiting 2 h after goserelin injection, dizziness, diplopia	Androgen deprivation therapy (goserelin) for prostate cancer	Pituitary macroadenoma
Alkhaibary [214] 2021	Case report	65-year-old male	Diplopia, 3rd cranial nerve palsy	Coronary bypass graft surgery	Pituitary adenoma
Ambrose [158] 2021	Case report	84-year-old male	Headache, bruising 5 days after admission: bilateral ptosis, ophthalmoplegia	Immune thrombocytopenia	Pituitary macroadenoma
Bhogal [215] 2021	Case report	63-year-old male	Myxedema coma		Pituitary adenoma
Bukhari [159] 2021	Case report	59-year-old male	Headache, loss of vision, 3rd cranial nerve palsy	Hypertension	Pituitary adenoma
Cavalli [160] 2021	Retrospective study	30 patients (22 males, 8 females) mean age: 54 years	Visual disturbance (86.7%) Headache (96%) Nausea and vomiting (33.3%)	Hypertension (53.8%) Invasive procedures (30.7%) Anticoagulation (23%) Trauma (7.7%) Antiplatelet therapy (7.7%)	PitNET
de Silva [161] 2021	Case report	35-year-old female	Headache, visual loss Cushingoid features		Crooke cell tumor
Elsehety [216] 2021	Case report	60-year-old female	Vision loss, vomiting, coma		Nonfunctioning pituitary adenoma
Falhammar [162] 2021	Retrospective study	33 patients (18 male and 15 female) mean age: 46.5 years	Headache (82%) Visual disturbances (36%) Nausea (36%)	Antithrombotic therapy *n* = 7 (21%)	PitNET
Gohil [163] 2021	Case report	56-year-old male	Headache, diplopia, 3rd cranial nerve palsy during hospitalization for leptospirosis	Leptospirosis	Pituitary adenoma
Hanna [164] 2021	Case report	49-year-old male	Headache, diplopia, 3rd cranial nerve palsy		Nonfunctioning pituitary adenoma
Huang [165] 2021	Case report	72-year-old male	Headache, nausea, vomiting, 3rd cranial nerve palsy		Nonfunctioning pituitary adenoma
Iqbal [166] 2021	Retrospective study	55 patients (26 males and 29 females) mean age: 50 years	Headache Confusion Visual field defect Cranial nerve palsy	Hypertension Anticoagulation Antiplatelet Previous stroke Radiotherapy	Somatotroph PitNET Lactotroph PitNET
Komić [167] 2021	Case report	54-year-old male	Headache, vomiting, photophobia		Pituitary adenoma
Marx [168] 2021	Retrospective study	46 patients (29 males and 17 females) mean age: 47.3 years	Meningism (*n* = 20) Headache (*n* = 38) Visual acuity decrease (*n* = 11) Visual field defect unilateral impairment bitemporal impairment (*n* = 20) Cranial nerve palsies: 3rd (*n* = 19), 4th (*n* = 6), 6th (*n* = 13) Impaired Glasgow Coma Scale ( < 15) (*n* = 2)	GnRH analogs (*n* = 2) Anticoagulant (*n* = 1) Cabergoline (*n* = 2) Recent major surgery (*n* = 3) Recent rugby play without head trauma (*n* = 1) Pregnancy (*n* = 3)	Nonfunctioning adenoma (*n* = 31) Corticotroph PitNET (*n* = 1) Lactotroph PitNET (*n* = 12) Somatotroph PitNET (*n* = 1)
Nakhleh [169] 2021	Retrospective study	27 patients (14 males and 13 females) mean age: 40.7 ± 12.5 years	Headache (*n* = 25) Visual field defect (*n* = 9) Cranial nerve palsy (*n* = 3) Altered consciousness (*n* = 2)	Hypertension (*n* = 5) DM (*n* = 3) Antiplatelet therapy (*n* = 3)	Nonfunctioning pituitary adenoma (*n* = 21) Lactotroph PitNET (*n* = 5) Somatotroph PitNET (*n* = 1)
Oudghiri [170] 2021	Case series	4 patients (2 males and 2 females) mean age: 78.25 years	Headache (*n* = 2) Vomiting (*n* = 2) Visual disturbance (*n* = 1) Cranial nerve palsy (*n* = 3)	Anticoagulant (*n* = 2) Hypertension (*n* = 1) DM (*n* = 1)	Nonfunctioning pituitary adenoma
Pan [171] 2021	Case report	44-year-old male	Headache, vomiting, dizziness	Diabetic ketoacidosis Acute pancreatitis Hypertension	Somatotroph PitNET
Pattankar [172] 2021	Case report	20-year-old female	Headache, lethargy, altered sensorium Progressive neurological deterioration: hemiparesis, unilateral ophthalmoplegia	Diabetic ketoacidosis	Nonfunctioning pituitary adenoma
Pokhrel [217] 2021	Case report	26-year-old male	Vision reduction, retro-orbital pain, dizziness, vomiting, right limbs weakness Acromegalic features	No apparent trigger	Somatotroph PitNET
Rosso [173] 2021	Case report	76-year-old female	Headache, 3rd cranial nerve palsy	2DM apixaban	Pituitary adenoma
Seaman [25] 2021	Retrospective study	44 patients (24 males and 20 females) median age: 55 years	Headache (*n* = 40) Visual disturbances (*n* = 30): visual field disturbances (*n* = 16), decreased visual acuity (*n* = 9), cranial nerve palsies: 3rd (*n* = 18), 4th (*n* = 6), 5th (*n* = 1), 6th (*n* = 6) Nausea/vomiting (*n* = 7) Altered mental status (*n* = 7) Hormone-related complains (*n* = 4)	Hypertension (*n* = 22) DM (*n* = 13) Anticoagulation (*n* = 4)	Nonfunctioning adenoma (*n* = 38) Functioning PitNET (*n* = 6)
Sun Z [174] 2021	Retrospective study	24 patients (13 males and 11 females) mean age: 46.46 ± 14.95 years	Headache (*n* = 20, 83.33%) Nausea and vomiting (*n* = 17, 70.83%) Loss of vision (*n* = 18, 75.00%) Visual field defects (*n* = 8, 33.33%) Ophthalmoplegia attributed mainly to 3rd and 6th cranial nerve palsies (*n* = 7, 29.17%) Decreased libido (*n* = 2, 8.33%) Amenorrhea (*n* = 2, 8.33%) Loss of consciousness (*n* = 1)		Nonfunctioning adenomas (*n* = 7) Lactotroph PitNET (*n* = 3) Corticotroph PitNET (*n* = 1) Gonadotroph PitNET (*n* = 1) Lactocorticotroph PitNET (*n* = 1)
Teramoto [175] 2021	Retrospective study	45 patients (33 males and 12 females) mean age: 56 years	Headache (*n* = 40) Visual impairment (*n* = 18) Ophthalmoplegia (*n* = 19) Hypopituitarism (*n* = 23)	Hypertension (*n* = 12) DM (*n* = 10) Antithrombotic therapy (*n* = 7)	Nonfunctioning adenoma (*n* = 37) Somatotroph PitNET (*n* = 5) Lactotroph PitNET (*n* = 3)
Tumyan [176] 2021	Case report	67-year-old male	Headache, nausea, peripheral vision defect, decreased consciousness within 48 h; 3rd cranial nerve palsy, progressive confusion	DM	Pituitary adenoma
Van Dong [177] 2021	Case report	38-year-old female	Headache, decreased visual acuity, aphasia, hemiparesis, decreased consciousness		Pituitary adenoma
Vargas [178] 2021	Case report	53-year-old female	Headache, vomiting, 3rd cranial nerve palsy, progressive neurological deterioration		Pituitary macroadenoma
Yoshida [179] 2021	Case report	78-year-old male	Headache, nausea, hemianopia, collapse		Gonadotroph PitNET
Zhu JD [180] 2021	Case report	48-year-old male	Headache, vomiting, 3rd cranial nerve palsy, decreased vision unilaterally within 48 h: consciousness disturbance, hemiplegia, hyperthermia		Nonfunctioning pituitary adenoma
Zhu Q [181] 2021	Retrospective study	46 patients (35 males and 11 females) mean age: 46.78 ± 12.32	Headache (*n* = 44) Visual disturbance (*n* = 41) Nausea/vomiting (*n* = 27) Hypogonadism (*n* = 39) Asymptomatic apoplexy (*n* = 1)		PitNET
Cross [182] 2022	Retrospective study	59 patients (40 males and 19 females) median age: 54 years	Headache (*n* = 59) Cranial nerve deficits (*n* = 46): 3rd cranial nerve palsy (*n* = 26, 44%), 4th cranial nerve palsy (*n* = 1), 6th cranial nerve paly (*n* = 12, 20%) Vision loss (*n* = 5)		PitNET
Geyik [183] 2022	Retrospective study	143 patients with pituitary adenoma, out of which 8 patients with PA (4 males and 4 females, mean age: 26.75 years)	Headache (*n* = 7) Visual disturbances (*n* = 4) Amenorrhea and galactorrhea (*n* = 4)		Lactotroph PitNET (*n* = 5) Nonfunctioning adenoma (*n* = 3)
Hamrick [184] 2022	Case report	31-year-old male	Headache, nausea, vision decrease,	Systemic chemotherapy (bleomycin, etoposide, cisplatin)	Somato- and corticotroph PitNET
Hsu [185] 2022	Case report	53-year-old male	Headache, fever, pruritic skin rash, abdominal pain, fatigue		Gonadotroph PitNET
Liu [186] 2022	Case report	33-year-old male	Headache, nausea, vomiting, bitemporal hemianopia, diplopia, 3rd cranial nerve palsy, Cushingoid features		Corticotroph PitNET
Mills [187] 2022	Case report	65-year-old female	Headache, reduced visual acuity, bitemporal hemianopia		Metastatic breast carcinoma into gonadotroph PitNET
Oldfield [188] 2022	Case report	57-year-old male	Headache, thirst, polydipsia, weakness		Pituitary adenoma
Puglisi [189] 2022	Case report	81-year-old female	Headache, bilateral vision loss, altered consciousness, hematuria	Anticoagulant (acenocoumarol), hypertension	PitNET
Rai [190] 2022	Case report	64-year-old male	Headache, nausea, vomiting, decreased visual acuity, ophthalmoplegia	Hypertension, DM, polysubstance abuse	Pituitary macroadenoma
Shrestha [191] 2022	2 case reports	56-year-old male	Headache, 3rd cranial nerve palsy, diplopia, decrease in vision	Hypertension	PitNET
		50-year-old male	3rd cranial nerve palsy (ptosis)		
Singh A. [192] 2022	Case report	36-year-old male	Chronic headache, sudden diplopia, 6th cranial nerve palsy		Somatotroph PitNET
Singh V. [193] 2022	Case report	52-year-old female	Headache, visual field changes		Nonfunctioning pituitary adenoma
Syed [194] 2022	Case report	36-year-old male	Headache, diplopia, abducens nerve palsy		Pituitary adenoma
Viola [195] 2022	Case report	63-year-old male	Headache, asthenia, diplopia		Pituitary adenoma

* there are some reports where imaging findings did not identify an actual pituitary tumor at the moment of PA confirmation. Of note, due to the changes of terminology during the last decade, we used the type of pituitary tumor according to the original papers unless there were clear criteria for what we currently consider PitNET. Abbreviations: BP = blood pressure; DM = diabetes mellitus; 2 DM = type 2 diabetes mellitus; GnRH = gonadotropin-releasing hormone; T3 = triiodothyronine; PA = pituitary apoplexy; *n* = number of patients (the name of the original article “study” or “series” was used according to the original publication).

**Table 2:** Management and outcome in patients PitNET–PA [29,30,31,32,33,34,35,36,37,38,39,40,41,42,43,44,45,46,47,48,49,50,51,52,53,54,55,56,57,58,59,60,61,62,63,64,65,66,67,68,69,70,71,72,73,74,75,76,77,78,79,80,81,82,83,84,85,86,87,88,89,90,91,92,93,94,95,96,97,98,99,100,101,102,103,104,105,106,107,108,109,110,111,112,113,114,115,116,117,118,119,120,121,122,123,124,125,126,127,128,129,130,131,132,133,134,135,136,137,138,139,140,141,142,143,144,145,146,147,148,149,150,151,152,153,154,155,156,157,158,159,160,161,162,163,164,165,166,167,168,169,170,171,172,173,174,175,176,177,178,179,180,181,182,183,184,185,186,187,188,189,190,191,192,193,194,195,196,197,198,199,200,201,202,203,204,205,206,207,208,209,210,211,212,213,214,215,216,217,218,219,220,221,222].

Author (Reference)	Management	Outcome
Brar [29]	Transfer to a lower altitude glucocorticoid replacement	Adrenal insufficiency
Cagnin [30]	C	Resolution of neurological symptoms, hypopituitarism
Chan [31]	TSS Hormonal replacement (postoperative hypopituitarism)	3 years follow-up: remission of the pituitary tumor, regression of Cushingoid features, permanent anterior hypopituitarism and central diabetes insipidus
Chentli [32]	C	2 months follow-up: visual acuity normalized, spontaneous reduction in pituitary tumor (MRI)
Choudhry [33]	TSS (*n* = 4) Hormonal replacement (postoperative hypopituitarism)	Postoperatively: vision recovery and biochemical remission of Cushing’s disease (*n* = 4) 6 months follow-up: regression of cushingoid features (*n* = 4), weight-loss (*n* = 4), hypertension remission (*n* = 3), DM resolution (*n* = 3), DM improvement (*n* = 1) 40 months follow-up: biochemical remission and postoperative anterior hypopituitarism (*n* = 4)
Enatsu [196]	TSS	3rd cranial nerve palsy improved Complete removal of the mass (MRI) Endocrine profile recovery
Komurcu [34]	TSS	4 months follow-up: complete visual recovery 6 months follow-up: complete tumor removal, no recurrence (CT)
Kruljac [35]	TSS	Exitus 1 month later
Kurisu [36]	Craniotomy, evacuation of the hemorrhage, tumor resection through anterior interhemispheric approach	Exitus within 1 month (PA causing intracerebral hemorrhage)
Liu [37]	C	3 months follow-up: normal pituitary hormones secretion, complete disappearance of pituitary mass (MRI)
Mohindra [38]	Ventriculo-peritoneal shunt	Exitus within 48 h
Paisley [39]	TSS Glucocorticoid replacement	Visual recovery, partial hypopituitarism
Tedd [40]	Glucocorticoid replacement	
Verma [41]	TSS Glucocorticoid replacement	Clinical improvement
Wildemberg [42]	TSS	
Yamamoto [43]	TSS Transient desmopressin replacement	Symptoms resolved postoperatively and vision returned
	TSS	Visual disturbances resolved + No hormonal deficits
Zoli [44]	TSS	3 months follow-up: radical tumor removal, resolution of neurological symptoms
Chou [45]	TSS	Resolution of visual disturbances
Cinar [46]	TSS	Remission of acromegaly
Delgado-Alvarado [47]	TSS	Resolution of ophthalmoplegia
Deshwal [48]	Transfer to a lower altitude + C	Full recovery
Fanous [49]	TSS	Near complete resolution of visual disturbances
Haider [50]	C (+ Dexamethasone)	Complete recovery of 3rd cranial nerve function
Hojo [51]	Initial C → TSS following hematoma expansion	No visual disturbances
Huang [52]	TSS	Vision improvement
Jiang [53]	C	Patient refused further treatment
Kobayashi [54]	C	Recovery without sequela
Machado [220]	C	28 months follow-up: clinical remission
Masui [55]	TSS	Anterior pituitary hormonal deficits, diabetes insipidus
Mir [56]	TSS Glucocorticoid replacement	Remission of DM; normalization of GH levels Central hypothyroidism, hypogonadism, and hypocortisolism
Mohamed [57]	TSS	Postoperatively: partial improvement of vision and 3rd cranial nerve palsy
Ní Chróinín [58]	C	Clinical improvement
Oh [59]	TSS	Clinical improvement
Radhiana [60]	Craniotomy, decompression, tumor excision	Exitus
Steinberg [218]	Patient refused surgery C (Cabergoline)	Tumor size decreased, necrotic zone in the pituitary (MRI)
Uemura [219]	C (Glucocorticoid, levothyroxine replacement)	Resolution of signs and symptoms
Witczak [62]	TSS	Exitus after 3 months (due to breast cancer)
Wong [63]	TSS	Recovery of nerve palsies
Zieliński [64]	C (Glucocorticoid, levothyroxine replacement)	Tumor regression (MRI); resolution of ophthalmic and neurological symptoms
Berkenstock [65]	TSS	Resolution of nerve palsy, persistent visual field defects, panhypopituitarism
Bujawansa [66]	Pituitary surgery (*n* = 33, 55%): early emergency surgery (*n* = 23), delayed elective surgery (*n* = 18) conservative (*n* = 22, 40%)	Complete resolution of visual field defects (65%) Partial improvement of visual field defects (85%) Complete resolution of cranial nerve palsies (*n* = 23) Partial resolution of cranial nerve palsies (*n* = 26) Anterior pituitary deficits (*n* = 47, 85.5%) Central adrenal insufficiency (*n* = 40, 72.7%) Central hypothyroidism (29 patients, 52.7%) Severe growth hormone deficiency (*n* = 21, 38.2%) Hypogonadism (*n* = 27 male patients) Diabetes insipidus (*n* = 2)
Chao [67]	TSS	Visual field improvement
Cho [68]	C	Partial recovery, no endocrine dysfunctions
Garg [197]	TSS	
Gupta [69]	Coronary artery bypass → TSS	Resolution of nerve palsy, persistent visual field defects
Jho [70]	TSS (*n* = 101) C (*n* = 8)	Resolution of symptoms (*n* = 48) Improvement of symptoms (*n* = 12) Stationary (*n* = 1)
Lee [71]	TSS Glucocorticoid replacement, anti-thyroid drugs	
Maltby [72]	C	Panhypopituitarism, empty sella
Man [73]	Palliative care, dexamethasone, brain radiotherapy	
Mishra [74]	TSS	Visual improvement
Mura [198]	Conservative	Hypogonadotrophic hypogonadism
Navarro-Bonnet [75]	Fronto-temporoparietal craniectomy	Postoperatively: hemiplegia, hormonal substitution (prednisone, levothyroxine), cabergoline treatment 6 months follow-up: amelioration of paralysis, normalized prolactin levels, small residual tumor
Rebeiz [199]	TSS	No improvement
Roerink [76]	C (Hydrocortisone, levothyroxine, lanreotide, scheduled TSS)	3 months follow-up: normalized IGF-1 levels
Tan [77]	C (Cabergoline, dexamethasone) → TSS due to visual deterioration	Hypopituitarism, persistent visual defects, residual tumor
Villar-Taibo [78]	C (Hormonal replacement)	Resolution of symptoms, improvement of acromegaly signs and symptoms, hypopituitarism, diabetes insipidus
Yoshida [200]	TSS Hormonal replacement	Hypopituitarism, resolution of hyperthyroidism, with subsequent hypothyroidism
Yoshino [201]	C (Glucocorticoid replacement)	Resolution of pituitary hemorrhage
Zhang [79]	TSS Hormonal replacement	Symptoms improvement
Akakın [80]	TSS Hormonal replacement	Visual defects improvement, no residual tumor
Asaithambi [81]	TSS	Postoperatively: bitemporal hemianopia, left and right partial 3rd cranial nerve palsy
Banerjee [82]	TSS	No improvement
Fountas [83]	TSS Hormonal replacement	Hypopituitarism
Kasl [202]	TSS	Improvement of neurological symptoms
Kasl [203]	TSS	Recovery of symptoms
Kim [84]	TSS	Complete recovery
Man [85]	TSS	Partial improvement of 3rd cranial nerve palsy
Roerink [86]	C	
	C → elective TSS	
Saberifard [87]	C	Empty sella
Sasagawa [88]	TSS	Complete resolution of nerve palsy
Sasaki [89]	C	
Singh [90]	Surgery during acute stage (*n* = 61, 70.1%) Delayed surgery (*n* = 8, 9.2%) Conservative (*n* = 18, 20.7%)	Improvement/complete resolution of visual defects in all survivors HRT with levothyroxine (62.7%) or cortisol (60%) Diabetes insipidus at 3-month follow-up (23%) Tumor regrowth (*n* = 7, 8.6%) Exitus (*n* = 4, 4.6%)
Teasdale [91]	TSS 5 years after the initial presentation: sphenoidotomy, radiotherapy	Visual improvement, panhypopituitarism 5 years after the initial presentation: exitus
Zhu [92]	TSS (*n* = 93, 95.88%) Transcranial surgery (*n* = 4, 4.12%)	Electrolyte disturbance (*n* = 18, 18.56%) Diabetes insipidus (*n* = 69, 71.13%) Cerebrospinal rhinorrhea (*n* = 2, 2.06%) Postoperative hemorrhage (*n* = 1, 1.03%) Postoperative infection (*n* = 1, 1.03%) Exitus (*n* = 1, 1.03%)
Zou [93]	Surgery	Improvement of symptoms and radiological findings
Choudhury [94]	C	Considerable reduction in pituitary tumor
Doglietto [95]	TSS	Recovery of nerve palsy, complete removal of pituitary adenoma (MRI)
Gambaracci [96]	TSS	
Giammattei [97]	TSS (*n* = 8) Hormonal replacement	Hypopituitarism
Giritharan [98]	C (*n* = 11, 35%) Surgery (*n* = 20, 65%)	Hormone deficiency (*n* = 26, 84%) Resolution of visual disturbances (*n* = 18, 72%) Improvement of visual symptoms (*n* = 6, 24%)
Keane [99]	TSS	Complete resolution of symptoms
Ogawa [100]	TSS (*n* = 43)	Resolution of neurological symptoms (*n* = 38) Persistence of nerve palsy (*n* = 3) Prolonged visual problems (*n* = 1) Exitus due to leukemia (*n* = 1)
Paschou [101]	TSS	Resolution of symptoms, central hypothyroidism
Sussman [102]	TSS	6 weeks follow-up: partial recovery of lower limb paresis, persistence of upper limb paresis, full recovery of 3rd and 4th cranial nerve palsies, partial 6th cranial nerve palsy
Balaparameswara Rao [103]	TSS	Improvement of vision Anterior hypopituitarism
Grangeon [104]	C	Headache resolution
Humphreys [105]	TSS (*n* = 5) Radiotherapy (*n* = 2)	Hypopituitarism treated with HR (*n* = 4)
Ishigaki [204]	TSS	Resolution of nerve palsy
Law-Ye [106]	C	Full recovery
Pasha [107]	TSS	Recovery of neurological deficits
Patra [108]	C	Remission of acromegaly and polycythemia
Rais [205]	C	
Simsek Bagir [109]	TSS	Remission of symptoms, central hypothyroidism and hypogonadism, GH deficiency
Souteiro [110]	C	Resolution of hypercortisolism, disappearance of the tumor (MRI)
Waqar [111]	TSS (*n* = 36) Conservative (*n* = 11)	Hypopituitarism (*n* = 45) Diabetes insipidus (*n* = 3)
Zoli [112]	TSS (*n* = 75)	Anterior hypopituitarism (*n* = 15, 20%) Diabetes insipidus (*n* = 4, 5.3%) Ophthalmoplegia resolution/improvement (71%) Visual symptoms improvement (85.5%) Conscious regain in both patients
Abbara [113]	C (*n* = 33/52) Surgery (*n* = 19/52)	Partial resolution of visual symptoms (*n* = 19/19) Full resolution of visual symptoms (*n* = 5/16) Hypopituitarism: long-term hypocortisolism (*n* = 27), hypothyroidism (*n* = 15) Diabetes insipidus (*n* = 2)
Bettag [114]	TSS	Partial improvement of hemiparesis, persistent visual loss
Fan [115]	C	Complete resolution of visual disturbances after 6 months
Hodgson [206]	Embolization, surgical resection	Reduction in pain
Jang [207]	TSS	Improvement of visual disturbances
Joo [116]	TSS	Full recovery
Kuzu [118]	TSS	Recovery of symptoms, panhypopituitarism
Myla [119]	TSS	Hormonal replacement
Raj [208]	C	Remission of central adrenal insufficiency
Ricciuti [120]	Surgery (*n* = 13) C (*n* = 4)	Symptoms resolved in up to 6 months Hormonal substitution (*n* = 6) Residual tumor (*n* = 6)
Rutkowski [121]	Surgery (*n* = 32, 100%)	Improvement of visual symptoms (77%) Resolution of visual symptoms (38%) Resolution of oculomotor palsies (81%) Partial hormone recovery following preoperative hypopituitarism 6 (21%) of 28
Salehi [209]	TSS	Complete recovery
Ward [210]	TSS Hormonal replacement	Stable vision, improved mental status
Yamada [122]	C	Panhypopituitarism
Almeida [123]	Surgery (*n* = 49) C (*n* = 18)	Last follow-up: Visual status: worsening (*n* = 1), stationary (*n* = 3), partial improvement (*n* = 12), complete recovery (*n* = 24) Cranial nerve palsies: partial improvement (*n* = 9), complete recovery (*n* = 17) Hormonal function: hypothyroidism (*n* = 3), hypocortisolism (*n* = 11), panhypopituitarism (*n* = 27), diabetes insipidus (*n* = 4)
Crisman [124]	TSS	Resolution of cranial nerve palsy
Dupont [125]	TSS	Slight regain of vision postoperatively
Ghalaenovi [126]	Lost during follow-up	The patient experienced spontaneous resolution of initial symptoms
Harju [127]	Craniotomy Glucocorticoid replacement	Persistent visual defects, anosmia, diabetes insipidus
Hosmann [128]	TSS (*n* = 72, 94.7%) Surgery with sub-frontal approach (*n* = 4, 5.3%)	Decreased visual acuity (12.3%) Visual field deficit (35.4%) Impaired eye movement (14.1%) Hormonal replacement (72.3%): hypothyroidism (61.3%), hypocortisolism (52.3%), hypogonadism (29.2%), permanent diabetes insipidus (9.2%), GH deficiency (4.6%)
Kirigin Biloš [129]	C	Improvement of 3rd cranial nerve deficit, diplopia, panhypopituitarism, partial empty sella
Krug [130]	TSS	Recovery of symptoms
Mittal [131]	C → TSS	
Naito [132]	C	Resolution of symptoms
Nioi [133]		Exitus
Pedro [134]	C	
Santos [135]	C	Resolution of symptoms, hypopituitarism
Sanz-Sapera [136]	C (Hormonal replacement)	Acromegaly remission
Singhal [137]	TSS	
Swaid [138]	TSS	1 month follow-up: persistence of 3rd cranial nerve palsy
Thomas [139]	C	Full recovery of ptosis
Uneda [140]	TSS	Improvement of nerve palsy
Wang [141]	TSS (*n* = 21)	Hypopituitarism requiring hormonal replacement (*n* = 6) Resolution of visual field defects (*n* = 13), improvement of visual field defects (*n* = 4) Resolution of visual acuity (*n* = 15), improvement of visual acuity (*n* = 2) Recovery of crania nerve palsies (*n* = 10/10) Residual tumor (*n* = 4) Hemiplegia (*n* = 1)
Waqar [142]	TSS	Resolution of nerve palsy
Ahn [211]	TSS	Neurological improvement; resolution of ICA stenosis
Brown [143]	TSS Radiotherapy	Lost to follow-up
Catarino [144]	TSS	Clinical improvement
Eichberg [145]	C → TSS 4 years later due to tumor recurrence	Resolution of symptoms No hormonal deficits
Elarjani [146]	TSS	Neurological improvement
Franzese [147]	TSS	Resolution of symptoms
Klimko [148]	TSS	Remission of acromegalic features
Lee [149]	C	Gradual improvement of headache and nerve palsies
Marzoughi [150]	C due to multiple comorbidities	Resolution of diplopia and 3rd cranial nerve palsy, central hypothyroidism
Pangal [151]	TSS (*n* = 50)	Headache improvement (87%) Vision loss improvement (86%) Resolution of cranial nerve palsy resolved (72%) Partial improvement of cranial nerve palsy (11%) Panhypopituitarism persistence (48%) Development of panhypopituitarism (6%)
Patel [152]	Stress dose corticosteroids, TSS	Resolution of symptoms, vision improvement
Romano [212]	TSS	Visual and functional improvement
Shetty [153]	TSS	
Siwakoti [154]	C	
van Boven [155]	C	
Yang [213]	C	
Alam [156]	No specific treatment	Acromegaly remission
Aljabri [157]	C	Tumor regression, restart of goserelin treatment without side effects, hormonal substitution (corticosteroids, levothyroxin)
Alkhaibary [214]	C	Visual acuity and nerve palsy improvement, hypoadrenalism, hypothyroidism
Ambrose [158]	C → surgical decompression	Minor neurological improvement
Bhogal [215]	C	Tumor size reduction
Bukhari [159]	Fronto-temporoparietal craniotomy for tumor excision	Panhypopituitarism
Cavalli [160]	Conservative *n* = 20 (66.7%) + 8 patients undergoing delayed elective surgery Emergency surgery *n* = 10 (33.3%) Hormonal replacement *n* = 28 (93.3%)	Resolution of visual acuity defects in 61% Resolution of visual field defects in 64% Resolution of cranial nerve palsies in 69% Poor endocrine outcomes with various degrees of hypopituitarism
de Silva [161]	TSS	Gradual improvement, normalization of cortisol levels
Elsehety [216]	TSS	Exitus
Falhammar [162]	Immediate pituitary surgery *n* = 3 Delayed pituitary surgery *n* = 8	Visual defect and nerve palsies improved Hormonal deficits did not regress 3 deaths of unrelated causes during follow-up
Gohil [163]	C	Resolution of visual disturbances and nerve palsy; regression of adenoma Persistent hypothyroidism and hypocortisolism
Hanna [164]	C	Resolution of symptoms within 4 days
Huang [165]	TSS	Resolution of visual disturbances and nerve palsy Hormonal replacement therapy
Iqbal [166]	TSS (9/33)	Deterioration of pituitary function *n* = 5 Improvement of pituitary function *n* = 13
Komić [167]	C (Hormonal replacement)	Resolution of symptoms after conservative treatment
Marx [168]	C (*n* = 27) TSS (*n* = 19)	Resolution of visual acuity defects (*n* = 10/11) Resolution of visual field defects (*n* = 16/17) Resolution of cranial nerve palsies (*n* = 21/23) Hormonal deficits: corticotropic (*n* = 17), thyrotropin (*n* = 24), gonadotropic (*n* = 20), somatotropin (*n* = 6) Residual tumor (*n* = 17) Tumor recurrence (*n* = 4)
Nakhleh [169]	C (*n* = 10) TSS (*n* = 17)	Corticotropic deficiency (*n* = 13) Secondary hypothyroidism (*n* = 8) Testosterone replacement therapy (*n* = 7) Central diabetes insipidus (*n* = 4) Cabergoline treatment (*n* = 2) Persistence of visual field defect (*n* = 2) Persistence of cranial nerve palsy (*n* = 2)
Oudghiri [170]	C (*n* = 3) TSS (*n* = 1)	Resolution of cranial nerve palsy; hormonal replacement therapy
Pan [171]	TSS	Central hypothyroidism and hypocortisolism
Pattankar [172]	C	Left hemiparesis
Pokhrel [217]	Emergency craniotomy and tumor excision	Significant improvement of neurological and visual disturbances
Rosso [173]	TSS	Resolution of the nerve palsy
Seaman [25]	TSS: within 24 h (*n* = 34) within 24–48 h (*n* = 3) after 7 days (*n* = 7)	All cranial nerve palsies were resolved Visual field disturbances resolved in 13/16 cases Visual acuity decrease resolved in 6/9 cases Hormonal deficits (unresolved/acquired): hypothyroidism (*n* = 11) hypocortisolism (*n* = 6) hypogonadism (low testosterone) (*n* = 3) panhypopituitarism (*n* = 11) transient diabetes insipidus (*n* = 8) permanent diabetes insipidus (*n* = 3)
Sun [174]	TSS	Total tumor resection (*n* = 21, 87.50%) Headache resolution in all patients Cranial nerve palsies: full recovery (*n* = 4); improvement (*n* = 3) Vision: returned to normal (*n* = 14); improvement (*n* = 3); stationary (*n* = 1) Resolution of hormonal deficits (*n* = 5)
Teramoto [175]	TSS	
Tumyan [176]	TSS	Significant improvement of neurological and visual symptoms Hormonal replacement therapy
Van Dong [177]	TSS	No residual tumor; transient central adrenal insufficiency Improvement of visual and neurological symptoms
Vargas [178]		Condition worsened, family withdrew care
Yoshida [179]	TSS	Residual tumor (MRI); visual acuity improvement
Zhu [180]	TSS	Resolution of neurological symptoms; persistence of visual disturbances
Zhu [181]	TSS (*n* = 45) C (*n* = 1)	Decrease visual acuity (*n* = 7) Visual field deficit (*n* = 3) Resolution of all ocular palsy Endocrine dysfunction: hypocortisolism (*n* = 26) hypothyroidism (*n* = 19) hypogonadism (*n* = 24)
Cross [182]	TSS	Cranial nerve palsies: resolution (72%), improvement without resolution (16%) Diabetes insipidus (*n* = 5) Exitus due to heparin induced thrombocytopenia (*n* = 1)
Geyik [183]	TSS	
Hamrick [184]	TSS	Resolution of visual disturbances, central hypothyroidism
Hsu [185]	TSS	Hypopituitarism; hormone replacement therapy
Liu [186]	TSS	Remission of hypercortisolism, hypopituitarism, HRT
Mills [187]	TSS	12 days postoperatively: exitus due to sepsis
Oldfield [188]	C	No residual tumor (MRI after 5 months)
Puglisi [189]	C	
Rai [190]	TSS	Improvement of visual symptoms
Shrestha [191]	TSS	Resolution of nerve palsy, residual tumor
Singh [192]	TSS	Resolution of 6th cranial nerve palsy; normal IGF-1 levels
Singh [193]	TSS	Remission of panhypopituitarism, normalization of visual field
Syed [194]	C	Resolution of pituitary adenoma and abducens palsy
Viola [195]	C	Panhypopituitarism requiring hormonal replacement; resolution of ophthalmologic and neurologic symptoms

Abbreviations: C = conservative approach; CT = computed tomography; DM = diabetes mellitus; GH = growth hormone; IGF-1 = insulin-like growth factors; MRI = magnetic resonance imaging; PA = pituitary apoplexy; TSS = transsphenoidal surgery.

**Table 3:** Retrospective studies with minimum 10 patients confirmed with PA/study according to our methodology [25,66,70,90,100,111,112,113,120,121,123,128,141,151,160,162,166,168,169,174,181,182,183].

Author Reference Number/ Year of Publication	Population	Underlying Pituitary Condition
Bujawansa [66] 2014	55 patients (35 males and 20 females) Mean age: 58.4 years	Nonfunctioning pituitary adenomas in 45 cases (82%), lactotroph PitNETs in 6 cases (11.5%), somatotroph PitNETs in 4 cases (7.2%), multiple endocrine neoplasia syndrome in 2 cases
Jho [70] 2014	109 patients (69 males and 40 females) mean age: 51 years	PitNET (*n* = 98) Rathke’s cyst (*n* = 8) Primitive neuroectodermal tumor (*n* = 1) Craniofaringioma (*n* = 1) Metastatic lung carcinoma (*n* = 1)
Singh [90] 2015	87 patients (55 males and 30 females) mean age: 50.9 years	Null cell (*n* = 18) Lactotroph PitNET (*n* = 8)
Ogawa [100] 2016	43 patients (30 males and 13 females) mean age: 56.67 years	Nonfunctioning pituitary adenoma (*n* = 29) Lactosomatotroph PitNET (*n* = 5) Lactotroph PitNET (*n* = 4) Thyrotroph PitNET (*n* = 3) Somatotroph PitNET (*n* = 1) Corticotroph PitNET (*n* = 1)
Waqar [111] 2017	47 patients with pituitary apoplexy (33 males and 14 females) mean age: 54 ± 15 years Patients were compared with 50 surgically treated patients with nonfunctioning pituitary adenomas	Pituitary adenoma
Zoli [112] 2017	75 patients (45 males and 30 females) mean age: 52.4 ± 16.2 years	Pituitary adenoma
Abbara [113] 2018	52 patients (25 males and 27 females) mean age: 46.7 years	Nonfunctioning adenoma or gonadotroph PitNET (*n* = 47) Lactotroph PitNET (*n* = 5)
Ricciuti [120] 2018	17 patients (12 males and 5 females) mean age: 58.76 years	
Rutkowski [121] 2018	32 patients (21 males and 11 females) mean age: 49 years	Nonfunctional adenoma (70%) Clinically hypersecreting adenoma (15%) Lactotroph PitNET Somatotroph PitNET Corticotroph PitNET
Almeida [123] 2019	67 patients (41 males and 26 females) mean age: 57.4 +/− 16.2 years	Pituitary adenoma
Hosmann [128] 2019	76 patients (53 males and 23 females) mean age: 53.7 +/-14.3 years	Clinically nonfunctioning PitNETs (81%): gonadotroph PitNET (37.9%), null-cell (29.3%), plurihormonal (8.6%), corticotroph (3.5%), somatotroph (1.7%) Clinically functioning PitNETs: lactotroph (10.4%), corticotroph (6.9%), somatotroph (1.7%)
Wang [141] 2019	21 patients (15 males and 6 females), with a mean age of 50.7 ± 15.0 years	Nonfunctioning pituitary adenoma (*n* = 18) Lactotroph PitNET (*n* = 2) Somatotroph PitNET (*n* = 1)
Pangal [151] 2020	50 patients (31 males, 19 females) mean age: 53 years	Pituitary adenoma
Cavalli [160] 2021	30 patients (22 males, 8 females) mean age: 54 years	PitNET
Falhammar [162] 2021	33 patients (18 male and 15 female) mean age: 46.5 years	PitNET
Iqbal [166] 2021	55 patients (26 males and 29 females) mean age: 50 years	Somatotroph PitNET Lactotroph PitNET
Marx [168] 2021	46 patients (29 males and 17 females) mean age: 47.3 years	Nonfunctioning adenoma (*n* = 31) Corticotroph PitNET (*n* = 1) Lactotroph PitNET (*n* = 12) Somatotroph PitNET (*n* = 1)
Nakhleh [169] 2021	27 patients (14 males and 13 females) mean age: 40.7 ± 12.5 years	Nonfunctioning pituitary adenoma (*n* = 21) Lactotroph PitNET (*n* = 5) Somatotroph PitNET (*n* = 1)
Seaman [25] 2021	44 patients (24 males and 20 females) median age: 55 years	Nonfunctioning adenoma (*n* = 38) Functioning PitNET (*n* = 6)
Sun Z [174] 2021	24 patients (13 males and 11 females) mean age: 46.46 ± 14.95 years	Nonfunctioning adenomas (*n* = 7) Lactotroph PitNET (*n* = 3) Corticotroph PitNET (*n* = 1) Gondadotroph PitNET (*n* = 1) Lactocorticotroph PitNET (*n* = 1)
Teramoto [175] 2021	45 patients (33 males and 12 females) mean age: 56 years	Nonfunctioning adenoma (*n* = 37) Somatotroph PitNET (*n* = 5) Lactotroph PitNET (*n* = 3)
Zhu Q [181] 2021	46 patients (35 males and 11 females) mean age:46.78 ± 12.32	PitNET
Cross [182] 2022	59 patients (40 males and 19 females) median age: 54 years	PitNET
Geyik [183] 2022	143 patients with pituitary adenoma, out of which 8 patients with PA (4 males and 4 females, mean age: 26.75 years)	Lactotroph PitNET (*n* = 5) Nonfunctioning adenoma (*n* = 3)

Abbreviations: PitNET = pituitary neuroendocrine tumor; PA = pituitary apoplexy; *n* = number of patients.

## Data Availability

Not applicable.

## Abbreviations

CT	computed tomography
CSF	cerebrospinal fluid
HIF-1α	hypoxia-inducing factor
HMGB1	high-mobility group box 1
HR	hormonal replacement
GH	growth hormone
IGF-1	insulin-like growth factor
MMP	matrix metalloproteinase
MRI	magnetic resonance imaging
PA	pituitary apoplexy
PAS	pituitary apoplexy score
PitNET	pituitary neuroendocrine tumor
PDE	phosphodiesterase
PTTG	pituitary tumor-transforming gene
TNF-α	tumor necrosis factor-α
TSS	trans-sphenoidal surgery
VEGF	vascular endothelial growth factor

## References

[1] Pearce J.M. (2015). On the Origins of Pituitary Apoplexy. Eur. Neurol..

[2] Bi W.L., Dunn I.F., Laws E.R. (2015). Pituitary apoplexy. Endocrine.

[3] Araujo-Castro M., Berrocal V.R., Pascual-Corrales E. (2020). Pituitary tumors: Epidemiology and clinical presentation spectrum. Hormones.

[4] Post K.D. (2014). Pituitary apoplexy: Is it one entity?. World Neurosurg..

[5] Barkhoudarian G., Kelly D.F. (2019). Pituitary Apoplexy. Neurosurg. Clin. N. Am..

[6] Vicente A., Lecumberri B., Gálvez M. (2013). Clinical practice guideline for the diagnosis and treatment of pituitary apoplexy. Endocrinol. Nutr..

[7] Jankowski P.P., Crawford J.R., Khanna P., Malicki D.M., Ciacci J.D., Levy M.L. (2015). Pituitary tumor apoplexy in adolescents. World Neurosurg..

[8] Glezer A., Bronstein M.D. (2015). Pituitary apoplexy: Pathophysiology, diagnosis and management. Arq. Bras. Endocrinol. Metabol..

[9] Gupta P., Dutta P. (2018). Landscape of Molecular Events in Pituitary Apoplexy. Front. Endocrinol..

[10] Shan B., Gerez J., Haedo M., Fuertes M., Theodoropoulou M., Buchfelder M., Losa M., Stalla G.K., Arzt E., Renner U. (2012). RSUME is implicated in HIF-1-induced VEGF-A production in pituitary tumour cells. Endocr.-Relat. Cancer.

[11] Araki T., Sangtian J., Ruanpeng D., Tummala R., Clark B., Burmeister L., Peterson D., Venteicher A.S., Kawakami Y. (2021). Acute elevation of interleukin 6 and matrix metalloproteinase 9 during the onset of pituitary apoplexy in Cushing’s disease. Pituitary.

[12] Okuda T., Fujita M., Kato A. (2019). Significance of Elevated HMGB1 Expression in Pituitary Apoplexy. Anticancer Res..

[13] Kreitschmann-Andermahr I., Siegel S., Carneiro R.W., Maubach J.M., Harbeck B., Brabant G. (2013). Headache and pituitary disease: A systematic review. Clin. Endocrinol..

[14] Suri H., Dougherty C. (2019). Presentation and Management of Headache in Pituitary Apoplexy. Curr. Pain Headache Rep..

[15] Hage R., Eshraghi S.R., Oyesiku N.M., Ioachimescu A.G., Newman N.J., Biousse V., Bruce B.B. (2016). Third, Fourth, and Sixth Cranial Nerve Palsies in Pituitary Apoplexy. World Neurosurg..

[16] Mavridis I., Meliou M., Pyrgelis E.-S. (2018). Presenting Symptoms of Pituitary Apoplexy. J. Neurol. Surg. Part A Central Eur. Neurosurg..

[17] Sarwar K.N., Huda M.S.B., Van De Velde V., Hopkins L., Luck S., Preston R., McGowan B., Carroll P.V., Powrie J.K. (2013). The Prevalence and Natural History of Pituitary Hemorrhage in Prolactinoma. J. Clin. Endocrinol. Metab..

[18] Li Y., Qian Y., Qiao Y., Chen X., Xu J., Zhang C., Wang W., Li J., Deng X. (2020). Risk factors for the incidence of apoplexy in pituitary adenoma: A single-center study from southwestern China. Chin. Neurosurg. J..

[19] Cinar N., Tekinel Y., Dagdelen S., Oruckaptan H., Soylemezoglu F., Erbas T. (2013). Cavernous sinus invasion might be a risk factor for apoplexy. Pituitary.

[20] Jahangiri A., Clark A.J., Han S.J., Kunwar S., Blevins L.S., Aghi M.K. (2013). Socioeconomic factors associated with pituitary apoplexy: Clinical article. J. Neurosurg..

[21] Goyal P., Utz M., Gupta N., Kumar Y., Mangla M., Gupta S., Mangla R. (2018). Clinical and imaging features of pituitary apoplexy and role of imaging in differentiation of clinical mimics. Quant. Imaging Med. Surg..

[22] Boellis A., Di Napoli A., Romano A., Bozzao A. (2014). Pituitary apoplexy: An update on clinical and imaging features. Insights Imaging.

[23] Vaphiades M.S. (2017). Pituitary Ring Sign Plus Sphenoid Sinus Mucosal Thickening: Neuroimaging Signs of Pituitary Apoplexy. Neuro-Ophthalmology.

[24] E Baldeweg S., Vanderpump M., Drake W., Reddy N., Markey A., Plant G.T., Powell M., Sinha S., Wass J. (2016). Society for endocrinology endocrine emergency guidance: Emergency management of pituitary apoplexy in adult patients. Endocr. Connect..

[25] Seaman S.C., Dougherty M.C., Zanaty M., Bruch L.A., Graham S.M., Greenlee J.D.W. (2021). Visual and Hormone Outcomes in Pituitary Apoplexy: Results of a Single Surgeon, Single Institution 15-Year Retrospective Review and Pooled Data Analysis. J. Neurol. Surg. Part B Skull Base.

[26] Tu M., Lu Q., Zhu P., Zheng W. (2016). Surgical versus non-surgical treatment for pituitary apoplexy: A systematic review and meta-analysis. J. Neurol. Sci..

[27] Goshtasbi K., Abiri A., Sahyouni R., Mahboubi H., Raefsky S., Kuan E.C., Hsu F.P., Cadena G. (2019). Visual and Endocrine Recovery Following Conservative and Surgical Treatment of Pituitary Apoplexy: A Meta-Analysis. World Neurosurg..

[28] Sahyouni R., Goshtasbi K., Choi E., Mahboubi H., Le R., Khahera A.S., Hanna G.K., Hatefi D., Hsu F.P., Bhandarkar N.D. (2019). Vision Outcomes in Early versus Late Surgical Intervention of Pituitary Apoplexy: Meta-Analysis. World Neurosurg..

[29] Brar K.S., Garg M.K. (2012). High altitude-induced pituitary apoplexy. Singap. Med. J..

[30] Cagnin A., Marcante A., Orvieto E., Manara R. (2012). Pituitary tumor apoplexy presenting as infective meningoencephalitis. Neurol. Sci. Off. J. Ital. Neurol. Soc. Ital. Soc. Clin. Neurophysiol..

[31] Chan D., Rong T.C., Dalan R. (2012). Cushing’s disease presenting with pituitary apoplexy. J. Clin. Neurosci. Off. J. Neurosurg. Soc. Australas..

[32] Chentli F., Bey A., Belhimer F., Azzoug S. (2012). Spontaneous resolution of pituitary apoplexy in a giant boy under 10 years old. J. Pediatr. Endocrinol. Metab..

[33] Choudhry O.J., Choudhry A.J., Nunez E.A., Eloy J.A., Couldwell W.T., Ciric I.S., Liu J.K. (2012). Pituitary tumor apoplexy in patients with Cushing’s disease: Endocrinologic and visual outcomes after transsphenoidal surgery. Pituitary.

[34] Komurcu H.F., Ayberk G., Ozveren M.F., Anlar O. (2012). Pituitary Adenoma Apoplexy Presenting with Bilateral Third Nerve Palsy and Bilateral Proptosis: A Case Report. Med. Princ. Pract..

[35] Kruljac I., Čerina V., Pećina H.I., Pažanin L., Matić T., Božikov V., Vrkljan M. (2012). Pituitary Metastasis Presenting as Ischemic Pituitary Apoplexy Following Heparin-induced Thrombocytopenia. Endocr. Pathol..

[36] Kurisu K., Kawabori M., Niiya Y., Ohta Y., Mabuchi S., Houkin K. (2012). Pituitary Apoplexy Manifesting as Massive Intracerebral Hemorrhage. Neurol. Med.-Chir..

[37] Liu S., Wang X., Liu Y.-H., Mao Q. (2012). Spontaneous disappearance of the pituitary macroadenoma after apoplexy: A case report and review of the literature. Neurol. India.

[38] Mohindra S., Savardekar A., Tripathi M., Garg R. (2012). Pituitary apoplexy presenting with pure third ventricular bleed: A neurosurgical image. Neurol. India.

[39] Paisley A.N., Syed A.A. (2012). Pituitary apoplexy masquerading as bacterial meningitis. Can. Med. Assoc. J..

[40] Tedd H., Tuckett J., Arun C., Dhar A. (2012). An unusual case of sudden onset headache due to pituitary apoplexy: A case report and review of the new UK guidelines. J. R. Coll. Physicians Edinb..

[41] Verma R., Singh S., Patil T.B. (2012). Thalamic infarction in pituitary apolplexy syndrome. BMJ Case Rep..

[42] Wildemberg L.E.A., Neto L.V., Niemeyer P., Gasparetto E.L., Chimelli L., Gadelha M.R. (2012). Association of dengue hemorrhagic fever with multiple risk factors for pituitary apoplexy. Endocr. Pract. Off. J. Am. Coll. Endocrinol. Am. Assoc. Clin. Endocrinol..

[43] Yamamoto T., Yano S., Kuroda J.-I., Hasegawa Y., Hide T., Kuratsu J.-I. (2012). Pituitary Apoplexy Associated with Endocrine Stimulation Test: Endocrine Stimulation Test, Treatment, and Outcome. Case Rep. Endocrinol..

[44] Zoli M., Mazzatenta D., Pasquini E., Ambrosetto P., Frank G. (2012). Cavernous sinus apoplexy presenting isolated sixth cranial nerve palsy: Case report. Pituitary.

[45] Chou H.-W., Chang H.-A., Huang S.-Y., Tzeng N.-S. (2013). Complex visual illusions in a patient with pituitary apoplexy. Gen. Hosp. Psychiatry.

[46] Cinar N.U., Metin Y., Dagdelen S., Ziyal I., Soylemezoglu F., Erbas T. (2013). Spontaneous remission of acromegaly after infarctive apoplexy with a possible relation to MRI and diabetes mellitus. Neuro Endocrinol. Lett..

[47] Delgado-Alvarado M., Riancho J., Riancho-Zarrabeitia L., Sedano M., Polo J., Berciano J. (2013). Oftalmoplejía completa unilateral sin pérdida de visión como forma de presentación de una apoplejía pituitaria. Rev. Clin. Esp..

[48] Deshwal R. (2013). Pituitary Apoplexy Masquerading as Acute Mountain Sickness. Wilderness Environ. Med..

[49] Fanous A.A., Quigley E.P., Chin S.S., Couldwell W.T. (2013). Giant necrotic pituitary apoplexy. J. Clin. Neurosci. Off. J. Neurosurg. Soc. Australas..

[50] Haider A.S., Rao P.J. (2013). A 64-year-old woman with dilated right pupil, nausea, and headache. Digit. J. Ophthalmol..

[51] Hojo M., Goto M., Miyamoto S. (2013). Chronic expanding pituitary hematoma without rebleeding after pituitary apoplexy. Surg. Neurol. Int..

[52] Huang T.-Y., Lin J.-P., Lieu A.-S., Chen Y.-T., Chen H.-S., Jang M.-Y., Shen J.-T., Wu W.-J., Huang S.-P., Juan Y.-S. (2013). Pituitary apoplexy induced by Gonadotropin-releasing hormone agonists for treating prostate cancer-report of first Asian case. World J. Surg. Oncol..

[53] Jiang H.-J., Hung W.-W., Hsiao P.-J. (2013). A case of acromegaly complicated with diabetic ketoacidosis, pituitary apoplexy, and lymphoma. Kaohsiung J. Med. Sci..

[54] Kobayashi J., Miyashita K., Tamanaha T., Kobayashi N., Iihara K., Nagatsuka K. (2013). Pituitary ischemic apoplexy in a young woman using oral contraceptives: A case report. J. Stroke Cerebrovasc. Dis. Off. J. Natl. Stroke Assoc..

[55] Masui K., Yonezawa T., Shinji Y., Nakano R., Miyamae S. (2013). Pituitary Apoplexy Caused by Hemorrhage From Pituitary Metastatic Melanoma: Case Report. Neurol. Medico-Chirurgica.

[56] Mir S.A., Masoodi S.R., Bashir M.I., Wani A.I., Farooqui K.J., Kanth B., Bhat A.R. (2013). Dissociated hypopituitarism after spontaneous pituitary apoplexy in acromegaly. Indian J. Endocrinol. Metab..

[57] Mohamed A.H., Rodrigues J., Bradley M.D., Nelson R.J. (2013). Retroclival subdural haematoma secondary to pituitary apoplexy. Br. J. Neurosurg..

[58] Ní Chróinín D., Lambert J. (2013). Sudden headache, third nerve palsy and visual deficit: Thinking outside the subarachnoid haemorrhage box. Age Ageing.

[59] Oh K., Kim J.-H., Choi J.-W., Kang J.-K., Kim S.-H. (2013). Pituitary Apoplexy Mimicking Meningitis. Brain Tumor Res. Treat..

[60] Radhiana H., O Syazarina S., Azura A.M.S., Azizi A.B. (2013). Pituitary apoplexy: A rare cause of middle cerebral artery infarction. Med. J. Malays..

[61] Tutanc M., Altas M., Yengil E., Ustun I., Dolapcioglu K.S., Balci A., Sefil F., Gokce C. (2013). Pituitary apoplexy due to thyroxine therapy in a patient with congenital hypothyroidism. Acta Med. Indones..

[62] Witczak J.K., Davies R., Okosieme O.E. (2013). An unusual case of pituitary apoplexy. QJM Mon. J. Assoc. Physicians.

[63] Wong S.H., Das K., Javadpour M. (2013). Pituitary apoplexy initially mistaken for bacterial meningitis. BMJ Case Rep..

[64] Zieliński G., Witek P., Koziarski A., Podgórski J. (2013). Spontaneous regression of non-functioning pituitary adenoma due to pituitary apoplexy following anticoagulation treatment—A case report and review of the literature. Endokrynol. Polska.

[65] Berkenstock M., Szeles A., Ackert J. (2014). Encephalopathy, Chiasmal Compression, Ophthalmoplegia, and Diabetes Insipidus in Pituitary Apoplexy. Neuro-Ophthalmology.

[66] Bujawansa S., Thondam S.K., Steele C., Cuthbertson D., Gilkes C.E., Noonan C., Bleaney C.W., Macfarlane I.A., Javadpour M., Daousi C. (2014). Presentation, management and outcomes in acute pituitary apoplexy: A large single-centre experience from the United Kingdom. Clin. Endocrinol..

[67] Chao C.C., Lin C.J. (2014). Pituitary apoplexy in a teenager—Case report. Pediatr. Neurol..

[68] Cho T.H., Rheims S., Ritzenthaler T., Berthezene Y., Nighoghossian N. (2014). Stroke and pituitary apoplexy revealing an internal carotid artery dissection. J. Stroke Cerebrovasc. Dis. Off. J. Natl. Stroke Assoc..

[69] Gupta V., Patil S., Raval D., Gopani P. (2014). Pituitary apoplexy presenting as myocardial infarction. Indian J. Endocrinol. Metab..

[70] Jho D.H., Biller B.M., Agarwalla P.K., Swearingen B. (2014). Pituitary Apoplexy: Large Surgical Series with Grading System. World Neurosurg..

[71] Lee K.A., Park T.S., Baek H.S., Jin H.Y. (2014). Pituitary apoplexy in T3 thyrotoxicosis. Endocrine.

[72] Maltby V.E., Crock P.A., Lüdecke D.K. (2014). A rare case of pituitary infarction leading to spontaneous tumour resolution and CSF-sella syndrome in an 11-year-old girl and a review of the pediatric literature. J. Pediatr. Endocrinol. Metab..

[73] Man B.L., Fu Y.P. (2014). Pituitary apoplexy in a patient with suspected metastatic bronchogenic carcinoma. BMJ Case Rep..

[74] Panigrahi S., Das S., Mishra S. (2014). Dengue hemorrhagic fever: A rare cause of pituitary apoplexy. Neurol. India.

[75] Navarro-Bonnet J., Anda J.J.M., Balderrama-Soto A., Pérez-Reyes S.P., Pérez-Neri I., Portocarrero-Ortiz L. (2014). Stroke associated with pituitary apoplexy in a giant prolactinoma: A case report. Clin. Neurol. Neurosurg..

[76] Roerink S., Marsman D., Van Bon A., Netea-Maier R. (2014). A Missed Diagnosis of Acromegaly During a Female-to-Male Gender Transition. Arch. Sex. Behav..

[77] Tan S.K., Seow C.J., Tan E., Chau Y.P., Dalan R. (2014). Pituitary apoplexy secondary to thrombocytopenia due to dengue hemorrhagic fever: A case report and review of the literature. Endocrine Practice: Official J. Am. Coll. Endocrinol. Am. Assoc. Clin. Endocrinol..

[78] Villar-Taibo R., Ballesteros-Pomar M.D., Vidal-Casariego A., Alvarez-San Martín R.M., Kyriakos G., Cano-Rodríguez I. (2014). Spontaneous remission of acromegaly: Apoplexy mimicking meningitis or meningitis as a cause of apoplexy?. Arq. Bras. Endocrinol. Metabol..

[79] Zhang C., Feng F., Zhu Y., Wang R., Xing B. (2014). Cerebral infarction caused by pituitary apoplexy: Case report and review of literature. Turk. Neurosurg..

[80] Akakın A., Yılmaz B., Ekşi M., Kılıç T. (2015). A case of pituitary apoplexy following posterior lumbar fusion surgery. J. Neurosurg. Spine.

[81] Asaithambi G. (2015). Carotid artery compression from pituitary apoplexy. QJM Int. J. Med..

[82] Banerjee C., Snelling B., Hanft S., Komotar R.J. (2015). Bilateral cerebral infarction in the setting of pituitary apoplexy: A case presentation and literature review. Pituitary.

[83] Fountas A., Andrikoula M., Tsatsoulis A. (2015). A 45 year old patient with headache, fever, and hyponatraemia. BMJ.

[84] Kim Y.H., Lee S.W., Son D.W., Cha S.H. (2015). Pituitary Apoplexy Following Mitral Valvuloplasty. J. Korean Neurosurg. Soc..

[85] Man B.L., Fu Y.P. (2015). Pituitary apoplexy presenting with bilateral oculomotor nerve palsy. BMJ Case Rep..

[86] Roerink S.H.P.P., Van Lindert E.J., Van De Ven A.C. (2015). Spontaneous remission of acromegaly and Cushing’s disease following pituitary apoplexy: Two case reports. Neth. J. Med..

[87] Saberifard J., Yektanezhad T., Assadi M. (2015). An Interesting Case of a Spontaneous Resolution of Pituitary Adenoma after Apoplexy. J. Belg. Soc. Radiol..

[88] Sasagawa Y., Tachibana O., Nakagawa A., Koya D., Iizuka H. (2015). Pituitary apoplexy following gonadotropin-releasing hormone agonist administration with gonadotropin-secreting pituitary adenoma. J. Clin. Neurosci..

[89] Sasaki Y., Nakata K., Suzuki K., Ando Y. (2015). Pituitary apoplexy presenting with anorexia and hyponatraemia. BMJ Case Rep..

[90] Singh T.D., Valizadeh N., Meyer F.B., Atkinson J.L.D., Erickson D., Rabinstein A.A. (2015). Management and outcomes of pituitary apoplexy. J. Neurosurg..

[91] Teasdale S., Hashem F., Olson S., Ong B., Inder W.J. (2015). Recurrent pituitary apoplexy due to two successive neoplasms presenting with ocular paresis and epistaxis. Endocrinol. Diabetes Metab. Case Rep..

[92] Zhu X., Wang Y., Zhao X., Jiang C., Zhang Q., Jiang W., Wang Y., Chen H., Shou X., Zhao Y. (2015). Incidence of Pituitary Apoplexy and Its Risk Factors in Chinese People: A Database Study of Patients with Pituitary Adenoma. PLoS ONE.

[93] Zou Z., Liu C., Sun B., Chen C., Xiong W., Che C., Huang H. (2015). Surgical treatment of pituitary apoplexy in association with hemispheric infarction. J. Clin. Neurosci..

[94] Choudhury M., Eligar V., DeLloyd A., Davies J.S. (2016). A case of pituitary apoplexy masquerading as subarachnoid hemorrhage. Clin. Case Rep..

[95] Doglietto F., Costi E., Villaret A.B., Mardighian D., Fontanella M., Giustina A. (2016). New oral anticoagulants and pituitary apoplexy. Pituitary.

[96] Gambaracci G., Rondoni V., Guercini G., Floridi P. (2016). Pituitary apoplexy complicated by vasospasm and bilateral cerebral infarction. BMJ Case Rep..

[97] Giammattei L., Mantovani G., Carrabba G., Ferrero S., Di Cristofori A., Verrua E., Guastella C., Pignataro L., Rampini P., Minichiello M. (2016). Pituitary apoplexy: Considerations on a single center experience and review of the literature. J. Endocrinol. Investig..

[98] Giritharan S., Gnanalingham K., Kearney T. (2016). Pituitary apoplexy—Bespoke patient management allows good clinical outcome. Clin. Endocrinol..

[99] Keane F., Egan A.M., Navin P., Brett F., Dennedy M. (2016). Gonadotropin-releasing hormone agonist-induced pituitary apoplexy. Endocrinol. Diabetes Metab. Case Rep..

[100] Ogawa Y., Niizuma K., Mugikura S., Tominaga T. (2016). Ischemic pituitary adenoma apoplexy—Clinical appearance and prognosis after surgical intervention. Clin. Neurol. Neurosurg..

[101] A Paschou S., Tzioras K., Trianti V., Lyra S., Lioutas V.-A., Seretis A., Vryonidou A. (2016). Young adult patient with headache, fever and blurred vision. Hormones.

[102] Sussman E.S., Ho A.L., Pendharkar A.V., Achrol A.S., Harsh G.R. (2016). Pituitary Apoplexy Associated with Carotid Compression and a Large Ischemic Penumbra. World Neurosurg..

[103] Arivazhagan A., Rao S.B., Savardekar A., Nandeesh B. (2017). Management dilemmas in a rare case of pituitary apoplexy in the setting of dengue hemorrhagic fever. Surg. Neurol. Int..

[104] Grangeon L., Moscatelli L., Zanin A., Guegan-Massardier E., Rouille A., Maltete D. (2017). Indomethacin-Responsive Paroxysmal Hemicrania in an Elderly Man: An Unusual Presentation of Pituitary Apoplexy. Headache.

[105] Humphreys G., Waqar M., McBain A., Gnanalingham K.K. (2017). Sphenoid sinus microbiota in pituitary apoplexy: A preliminary study. Pituitary.

[106] Law-Ye B., Pyatigorskaya N., Leclercq D. (2017). Pituitary Apoplexy Mimicking Bacterial Meningitis with Intracranial Hypertension. World Neurosurg..

[107] Pasha S.A., Ranganthan L.N., Setty V.K., Reddy R., Ponnuru D.A. (2017). Acute Ischaemic Stroke as a Manifestation of Pituitary Apoplexy in a Young Lady. J. Clin. Diagn. Res..

[108] Patra S., Biswas S.N., Datta J., Chakraborty P.P. (2017). Hypersomatotropism induced secondary polycythaemia leading to spontaneous pituitary apoplexy resulting in cure of acromegaly and remission of polycythaemia: ‘The virtuous circle’. BMJ Case Rep..

[109] Simsek Bagir G., Civi S., Kardes O., Kayaselcuk F., Ertorer M.E. (2017). Stubborn hiccups as a sign of massive apoplexy in a naive acromegaly patient with pituitary macroadenoma. Endocrinol. Diabetes Metab. Case Rep..

[110] Souteiro P., Belo S., Carvalho D. (2017). A rare case of spontaneous Cushing disease remission induced by pituitary apoplexy. J. Endocrinol. Investig..

[111] Waqar M., McCreary R., Kearney T., Karabatsou K., Gnanalingham K.K. (2017). Sphenoid sinus mucosal thickening in the acute phase of pituitary apoplexy. Pituitary.

[112] Zoli M., Milanese L., Faustini-Fustini M., Guaraldi F., Asioli S., Zenesini C., Righi A., Frank G., Foschini M.P., Sturiale C. (2017). Endoscopic Endonasal Surgery for Pituitary Apoplexy: Evidence On a 75-Case Series From a Tertiary Care Center. World Neurosurg..

[113] Abbara A., Clarke S., Eng P.C., Milburn J., Joshi D., Comninos A.N., Ramli R., Mehta A., Jones B., Wernig F. (2018). Clinical and biochemical characteristics of patients presenting with pituitary apoplexy. Endocr. Connect..

[114] Bettag C., Strasilla C., Steinbrecher A., Gerlach R. (2018). Unilateral Tuberothalamic Artery Ischemia Caused by Pituitary Apoplexy. J. Neurol. Surg. Part A Central Eur. Neurosurg..

[115] Fan Y., Bao X., Wang R. (2018). Conservative treatment cures an elderly pituitary apoplexy patient with oculomotor paralysis and optic nerve compression: A case report and systematic review of the literature. Clin. Interv. Aging.

[116] Joo C., Ha G., Jang Y. (2018). Pituitary apoplexy following lumbar fusion surgery in prone position: A case report. Medicine.

[117] Komshian S.R., Saket R., Bakhadirov K. (2018). Pituitary Apoplexy With Bilateral Oculomotor Nerve Palsy. Neurohospitalist.

[118] Kuzu F., Unal M., Gul S., Bayraktaroglu T. (2018). Pituitary Apoplexy due to the Diagnostic Test in a Cushing’s Disease Patient. Turk. Neurosurg..

[119] Myla M., Lewis J., Beach A., Sylejmani G., Burge M.R. (2018). A Perplexing Case of Pituitary Apoplexy Masquerading as Recurrent Meningitis. J. Investig. Med. High Impact Case Rep..

[120] Ricciuti R., Nocchi N., Arnaldi G., Polonara G., Luzi M. (2018). Pituitary adenoma apoplexy: Review of personal series. Asian J. Neurosurg..

[121] Rutkowski M.J., Kunwar S., Blevins L., Aghi M.K. (2018). Surgical intervention for pituitary apoplexy: An analysis of functional outcomes. J. Neurosurg..

[122] Yamada D., Fujikawa T. (2018). Pituitary apoplexy. Can. Med. Assoc. J..

[123] Almeida J.P., Sanchez M.M., Karekezi C., Warsi N., Fernández-Gajardo R., Panwar J., Mansouri A., Suppiah S., Nassiri F., Nejad R. (2019). Pituitary Apoplexy: Results of Surgical and Conservative Management Clinical Series and Review of the Literature. World Neurosurg..

[124] Crisman C., Ward M., Majmundar N., Damodara N., Hsueh W.D., Eloy J.A., Liu J.K. (2019). Pituitary Apoplexy Following Endoscopic Retrograde Cholangiopancreatography. World Neurosurg..

[125] Dupont G., Lachkar S., Iwanaga J., Tubbs R.S., Ishak B. (2019). Sudden Headache and Blindness Due to Pituitary (Adenoma) Infarction: A Case Report. Cureus.

[126] Ghalaenovi H., Azar M., Fattahi A. (2019). Spontaneous regression of nonfunctioning pituitary adenoma. Br. J. Neurosurg..

[127] Harju T., Alanko J., Numminen J. (2019). Pituitary apoplexy following endoscopic nasal surgery: A case report. SAGE Open Med. Case Rep..

[128] Hosmann A., Micko A., Frischer J.M., Roetzer T., Vila G., Wolfsberger S., Knosp E. (2019). Multiple Pituitary Apoplexy—Cavernous Sinus Invasion as Major Risk Factor for Recurrent Hemorrhage. World Neurosurg..

[129] Kirigin Biloš L.S., Kruljac I., Radošević J.M., Ćaćić M., Škoro I., Čerina V., Pećina I.H., Vrkljan M. (2019). Empty Sella in the Making. World Neurosurg..

[130] Krug R.G., Chang A.Y., Raghunathan A., Van Gompel J.J. (2019). Apoplectic Silent Crooke Cell Adenoma with Adjacent Pseudoaneurysms: Causation or Bystander?. World Neurosurg..

[131] Mittal A., Mishra S., Yadav K., Rajput R. (2019). Uncontrolled diabetes as a rare presenting cause of pituitary apoplexy. BMJ Case Rep..

[132] Naito Y., Mori J., Tazoe J., Tomida A., Yagyu S., Nakajima H., Iehara T., Tatsuzawa K., Mukai T., Hosoi H. (2019). Pituitary apoplexy after cardiac surgery in a 14-year-old girl with Carney complex: A case report. Endocr. J..

[133] Nioi M., Napoli P.E., Ferreli F. (2019). Fatal Iatrogenic Pituitary Apoplexy after Surgery for Neuroophthalmological Disorder. Anesthesiology.

[134] Pedro B., Patrícia T., Aldomiro F. (2019). Pituitary Apoplexy May Be Mistaken for Temporal Arteritis. Eur. J. Case Rep. Intern. Med..

[135] dos Santos A.R.M., Bello C.T., Sousa A., Duarte J.S., Campos L.B. (2019). Pituitary Apoplexy Following Systemic Anticoagulation. Eur. J. Case Rep. Intern. Med..

[136] Sanz-Sapera E., Sarria-Estrada S., Arikan F., Biagetti B. (2019). Acromegaly remission, SIADH and pituitary function recovery after macroadenoma apoplexy. Endocrinol. Diabetes Metab. Case Rep..

[137] Singhal A., Gohlke P.R., Chapman P.R. (2019). Spontaneous “pneumo-apoplexy” as a presentation of pituitary adenoma. Clin. Imaging.

[138] Swaid B., Kalaba F., Bachuwa G., Sullivan S.E. (2019). Heparin-Induced Pituitary Apoplexy Presenting as Isolated Unilateral Oculomotor Nerve Palsy: A Case Report and Literature Review. Case Rep. Endocrinol..

[139] Thomas M., Robert A., Rajole P., Robert P. (2019). A Rare Case of Pituitary Apoplexy Secondary to Dengue Fever-induced Thrombocytopenia. Cureus.

[140] Uneda A., Hirashita K., Yunoki M., Yoshino K., Date I. (2019). Pituitary adenoma apoplexy associated with vardenafil intake. Acta Neurochir..

[141] Wang Z., Gao L., Wang W., Guo X., Feng C., Lian W., Li Y., Xing B. (2019). Coagulative necrotic pituitary adenoma apoplexy: A retrospective study of 21 cases from a large pituitary center in China. Pituitary.

[142] Waqar M., Karabatsou K., Kearney T., Roncaroli F., Gnanalingham K.K. (2019). Classical pituitary apoplexy. Br. J. Hosp. Med..

[143] Brown T.V., Post K.D., Cheesman K.C. (2020). Recurrent Pituitary Apoplexy In An Adenoma With Switching Phenotypes. AACE Clin. Case Rep..

[144] Catarino D., Ribeiro C., Gomes L., Paiva I. (2020). Corticotroph adenoma and pituitary fungal infection: A rare association. Endocrinol. Diabetes Metab. Case Rep..

[145] Eichberg D.G., Di L., Shah A.H., Kaye W.A., Komotar R.J. (2018). Spontaneous preoperative pituitary adenoma resolution following apoplexy: A case presentation and literature review. Br. J. Neurosurg..

[146] Elarjani T., Chen S., Cajigas I., Saway B., Sur S., Morcos J.J. (2020). Pituitary Apoplexy and Cerebral Infarction: Case Report and Literature Review. World Neurosurg..

[147] Franzese I., Giambruno V., Tropea I., Linardi D., Petrilli G., Faggian G. (2020). Urgent Surgery for Pituitary Adenoma Bleeding After Coronary Bypass Surgery. Ann. Thorac. Surg..

[148] Klimko A., Capatina C. (2020). Pituitary Macroadenoma Presenting as Acromegaly and Subacute Pituitary Apoplexy: Case Report and Literature Review. Cureus.

[149] Lee I.H., Kim H.K., Ahn D.J. (2020). Concurrent pituitary apoplexy and posterior reversible encephalopathy syndrome in a patient with end-stage renal disease on hemodialysis: A case report. Medicine.

[150] Marzoughi S., Ganesh A., Qaddoura A., Motazedian P., Bal S.S. (2020). Pearls & Oy-sters: Isolated oculomotor nerve palsy due to pituitary apoplexy missed on CT scan. Neurology.

[151] Pangal D.J., Chesney K., Memel Z., Bonney P.A., Strickland B.A., Carmichael J., Shiroishi M., Liu C.-S.J., Zada G. (2020). Pituitary Apoplexy Case Series: Outcomes After Endoscopic Endonasal Transsphenoidal Surgery at a Single Tertiary Center. World Neurosurg..

[152] Patel A., Mobley B.C., Jagasia M., Adetola K., Byrne M., Dholaria B. (2020). Pituitary Apoplexy During Hematopoietic Cell Transplantation. Clin. Lymphoma Myeloma Leuk..

[153] Shetty S., Gnanaraj J., Roshan S.J., El Accaoui R. (2020). Pituitary apoplexy after regadenoson myocardial perfusion scan. J. Nucl. Cardiol..

[154] Siwakoti K., Omay S.B., Inzucchi S.E. (2020). Spontaneous Resolution of Primary Hypercortisolism of Cushing Disease After Pituitary Hemorrhage. AACE Clin. Case Rep..

[155] van Boven E., Massolt E.T., van Rossum E.F.C., Kiewiet-Kemper R.M. (2020). Spontaneous remission of unidentified Cushing’s disease revealed by hair cortisol analysis. Neth. J. Med..

[156] Alam S., Kubihal S., Goyal A., Jyotsna V.P. (2021). Spontaneous Remission of Acromegaly After Pituitary Apoplexy in a Middle-Aged Male. Ochsner J..

[157] Aljabri B., Lilleby W., Switlyk M.D., Tafjord G. (2021). Restart of androgen deprivation therapy after goserelin induced pituitary apoplexy in a patient with disseminated prostate cancer a case report and five-years follow-up. Urol. Case Rep..

[158] Ambrose C., Sarma S., Banerjee R., Myers S. (2021). Pituitary apoplexy and associated cranial nerve palsies secondary to bleeding caused by immune thrombocytopaenia in a patient with known pituitary macroadenoma. BMJ Case Rep..

[159] Bukhari K., Sharma V., Gupta S., Motazedi A. (2021). The snowman sign in a patient with pituitary tumor apoplexy. J. Community Hosp. Intern. Med. Perspect..

[160] Cavalli A., Martin A., Connolly D.J., Mirza S., Sinha S. (2021). Pituitary apoplexy: How to define safe boundaries of conservative management? Early and long-term outcomes from a single UK tertiary neurosurgical unit. Br. J. Neurosurg..

[161] de Silva N.L., Somasundaram N., Constantine R., Kularatna H. (2021). Apoplexy of Crooke cell tumour leading to the diagnosis of severe Cushing disease; a case report. BMC Endocr. Disord..

[162] Falhammar H., Tornvall S., Höybye C. (2021). Pituitary Apoplexy: A Retrospective Study of 33 Cases From a Single Center. Front. Endocrinol..

[163] Gohil J., Gowda A., George T., Easwer H.V., George A., Nair P. (2021). Pituitary apoplexy and panhypopituitarism following acute leptospirosis. Pituitary.

[164] Hanna V., Mednick Z., Micieli J. (2021). Rapid resolution of a third nerve palsy from pituitary apoplexy. BMJ Case Rep..

[165] Huang H., Jiang S., Yang C., Deng K., Wang R., Bao X. (2021). Surgical treatment of a 72-year-old patient with headache, hyponatremia and oculomotor nerve palsy: A case report and literature review. Gland. Surg..

[166] Iqbal F., Adams W., Dimitropoulos I., Muquit S., Flanagan D. (2021). Pituitary hemorrhage and infarction: The spectrum of disease. Endocr. Connect..

[167] Komić L., Kruljac I., Mirošević G., Gaćina P., Pećina H.I., Čerina V., Gajski D., Blaslov K., Rotim K., Vrkljan M. (2021). Spontaneous Resolution of a Nonfunctioning Pituitary Adenoma over One-Month Period: A Case Report. Acta Clin. Croat..

[168] Marx C., Rabilloud M., Borson Chazot F., Tilikete C., Jouanneau E., Raverot G. (2012). A key role for conservative treatment in the management of pituitary apoplexy. Endocrine.

[169] Nakhleh A., Assaliya Naffa M., Sviri G., Shehadeh N., Hochberg I. (2021). Outcomes of pituitary apoplexy: A comparison of microadenomas and macroadenomas. Pituitary.

[170] Oudghiri M.D., Motaib I., Elamari S., Laidi S., Chadli A. (2021). Pituitary Apoplexy in Geriatric Patients: A Report of Four Cases. Cureus.

[171] Pan J., Yang X., Zhu W. (2021). Domino effect of pituitary growth hormone tumor complicated by diabetic ketoacidosis and pituitary apoplexy: A case report. BMC Endocr. Disord..

[172] Pattankar S., Chauhan P., Kapadia F., Sankhe M. (2021). Pituitary apoplexy following severe diabetic ketoacidosis, with two uncommon complications of supraventricular tachycardia and acute limb ischemia, in a patient with neglected pituitary adenoma and undiagnosed diabetes mellitus: A rare clinical association. Asian J. Neurosurg..

[173] Rosso M., Ramaswamy S., Sucharew H., Vagal A., Anziska Y., Levine S.R. (2021). Isolated Third Cranial Nerve Palsy in Pituitary Apoplexy: Case Report and Systematic Review. J. Stroke Cerebrovasc. Dis..

[174] Sun Z., Cai X., Li Y., Shao D., Jiang Z. (2021). Endoscopic Endonasal Transsphenoidal Approach for the Surgical Treatment of Pituitary Apoplexy and Clinical Outcomes. Technol. Cancer Res. Treat..

[175] Teramoto S., Tahara S., Kondo A., Morita A. (2021). Key Factors Related to Internal Carotid Artery Stenosis Associated with Pituitary Apoplexy. World Neurosurg..

[176] Tumyan G., Mantha Y., Gill R., Feldman M. (2021). Acute Sterile Meningitis as a Primary Manifestation of Pituitary Apoplexy. AACE Clin. Case Rep..

[177] Van Dong H., Tran D., Chu H.T., Pham A.H., Nguyen X.T., Duong H.D. (2021). Emergency endoscopic surgery for pituitary apoplexy presenting as cerebral infarction in a limited resources condition: A case report. Int. J. Surg. Case Rep..

[178] Vargas A., Testai F.D. (2021). Pituitary Apoplexy Causing Bilateral Internal Carotid Artery Ischemia. Can. J. Neurol. Sci. J. Can. Sci. Neurol..

[179] Yoshida M., Hiu T., Baba S., Morikawa M., Horie N., Ujifuku K., Yoshida K., Matsunaga Y., Niino D., Xie A. (2021). Ruptured aneurysm–induced pituitary apoplexy: Illustrative case. J. Neurosurg. Case Lessons.

[180] Zhu J.-D., Xie S., Xu L., Xie M.-X., Xiao S.-W. (2021). The surgical management of pituitary apoplexy with occluded internal carotid artery and hidden intracranial aneurysm: Illustrative case. J. Neurosurg. Case Lessons.

[181] Zhu Q., Liang Y., Fan Z., Liu Y., Zhou C., Zhang H., Li T., Zhou Y., Yang J., Wang Y. (2022). Ischemic Infarction of Pituitary Apoplexy: A Retrospective Study of 46 Cases From a Single Tertiary Center. Front. Neurosci..

[182] Cross K.A., Desai R., Vellimana A., Liu Y., Rich K., Zipfel G., Dacey R., Chicoine M., Klatt-Cromwell C., McJunkin J. (2022). Surgery for Pituitary Tumor Apoplexy Is Associated with Rapid Headache and Cranial Nerve Improvement. Curr. Oncol..

[183] Geyik A.M., Durmaz M.O., Dogan A., Ugur B.K., Geyik S., Erkutlu I., Yasar S., Kırık A., Kose G., Nehir A. (2022). Pituitary Apoplexy: An Emergent and Potential Life-Threatening Complication of Pituitary Adenomas. Turk. J. Trauma Emerg. Surg..

[184] Hamrick F.A., Findlay M.C., Rennert R.C., Budohoski K.P., Couldwell W.T. (2022). Pituitary Apoplexy Precipitated by Systemic Chemotherapy. Cureus.

[185] Hsu C.C., Lin H.D., Huang C.Y., Chiang Y.L. (2022). Unusual manifestations of adrenal insufficiency: A case report of hypopituitarism and Well’s syndrome after apoplexy of a silent pituitary gonadotropic adenoma. Medicine.

[186] Liu T., Rossiter J.P., Houlden R.L., Awad S. (2022). Sparsely Granulated Corticotroph Pituitary Macroadenoma Presenting With Pituitary Apoplexy Resulting in Remission of Hypercortisolism. AACE Clin. Case Rep..

[187] Mills M.T., Wharton S.B., Connolly D.J., Mirza S., Sinha S. (2022). Pituitary apoplexy secondary to metastatic breast carcinoma into a gonadotroph cell adenoma of the pituitary. Br. J. Neurosurg..

[188] Oldfield E.H., Merrill M.J. (2015). Apoplexy of pituitary adenomas: The perfect storm. J. Neurosurg..

[189] Puglisi V., Morini E., Biasini F., Vinciguerra L., Lanza G., Bramanti P. (2022). Neurological Presentation of Giant Pituitary Tumour Apoplexy: Case Report and Literature Review of a Rare but Life-Threatening Condition. J. Clin. Med..

[190] Rai R.S., Gelnick S., Pomeranz H., Verma R. (2022). Recovery of Complete Blindness and Internal Ophthalmoplegia After Transsphenoidal Decompression of Pituitary Apoplexy. Cureus.

[191] Shrestha R., Bishokarma S., Rayamajhi S., Shrestha S., Lamichhane S., Shrestha P., Thulung S. (2022). Pituitary apoplexy presenting as isolated third cranial nerve palsy: Case series. J. Surg. Case Rep..

[192] Singh A., Khurana M., Pal H., Azad S., Sihag R.K., Kumar B. (2022). Bilateral sixth cranial nerve palsy, the first presenting feature of hemorrhagic apoplexy of pituitary macroadenoma: A case report. Int. J. Surg. Case Rep..

[193] Singh V., Holmes R. (2022). Visual recovery following surgical intervention for pituitary apoplexy correlated with preoperative optical coherence tomography. N. Z. Med. J..

[194] Syed S.B., Mourra A.A., Chatterjee T. (2022). Isolated Unilateral Abducens Nerve Palsy Manifesting as a Rare Complication of Idiopathic Pituitary Apoplexy: A Case Report. Cureus.

[195] Viola N., Urbani C., Cosottini M., Abruzzese A., Manetti L., Cosentino G., Marconcini G., Marcocci C., Bogazzi F., Lupi I. (2022). An altered state of consciousness while using anticoagulants and the incidental discovery of a pituitary lesion: Considering pituitary apoplexy. Endocrinol. Diabetes Metab. Case Rep..

[196] Enatsu R., Asahi M., Matsumoto M., Hirai O. (2012). Pituitary Apoplexy Presenting Atypical Time Course of Ophthalmic Symptoms. Tohoku J. Exp. Med..

[197] Garg M.K., Pathak H.C., Singh G. (2014). Subclinical pituitary apoplexy with preserved pituitary functions. Indian J. Endocrinol. Metab..

[198] Mura P., Cossu A.P., Musu M., De Giudici L.M., Corda L., Zucca R., Finco G. (2014). Pituitary apoplexy after laparoscopic surgery: A case report. Eur. Rev. Med. Pharmacol. Sci..

[199] Rebeiz T., Cueva W., Ardelt A. (2014). Unusual Case of Bilateral Caudate Infarcts Following Pituitary Apoplexy. JAMA Neurol..

[200] Yoshida M., Murakami M., Ueda H., Miyata M., Takahashi N., Oiso Y. (2014). An unusual case of hypopituitarism and transient thyrotoxicosis following asymptomatic pituitary apoplexy. Neuro Endocrinol. Lett..

[201] Yoshino M., Sekine Y., Koh E., Hata A., Hashimoto N. (2014). Pituitary Apoplexy After Surgical Treatment of Lung Cancer. Ann. Thorac. Surg..

[202] Kasl R.A., Hughes J., Burrows A.M., Meyer F.B. (2015). Pediatric ischemic stroke from an apoplectic prolactinoma. Child’s Nerv. Syst..

[203] Kistka H.M., Turner J.H., Devin J.K., Chambless L.B., Kasl R.A. (2015). Pituitary Apoplexy After Intravitreal Injection of Vascular Endothelial Growth Factor Inhibitor: A Novel Complication. J. Neurol. Surg. Rep..

[204] Ishigaki T., Kitano Y., Nishikawa H., Mouri G., Shimizu S., Miya F., Suzuki H. (2017). Delayed Onset of Isolated Unilateral Oculomotor Nerve Palsy Caused by Post-Traumatic Pituitary Apoplexy: A Case Report. Clin. Med. Insights Case Rep..

[205] Rais N.C., Merchant R.A., Seetharaman S.K. (2017). Pituitary apoplexy masquerading as functional decline in an older person. Age Ageing.

[206] Hodgson N.M., Campbell A.A., Chang J.R., Vizcaino A., Eberhart C., Pearl M.S., McCulley T.J. (2018). Pituitary Adenoma Apoplexy of the Orbit, Diagnosis, and Management With Presurgical Embolization. Ophthalmic Plast. Reconstr. Surg..

[207] Jang J.-H., Ko Y.S., Hong E.K., Gwak H.-S. (2018). Extensive Pituitary Apoplexy after Chemotherapy in a Patient with Metastatic Breast Cancer. Brain Tumor Res. Treat..

[208] Raj H., Kamalanathan S., Sahoo J.P., Kadhiravan T. (2018). Varicella causing remission of Cushing’s disease. BMJ Case Rep..

[209] Salehi N., Firek A., Munir I. (2018). Pituitary Apoplexy Presenting as Ophthalmoplegia and Altered Level of Consciousness without Headache. Case Rep. Endocrinol..

[210] Ward M., Kamal N., Majmundar N., de Leon A.B., Eloy J.A., Liu J.K. (2018). Post-Traumatic Pituitary Tumor Apoplexy After Closed Head Injury: Case Report and Review of the Literature. World Neurosurg..

[211] Ahn J.-M., Oh H.-J., Oh J.-S., Yoon S.-M. (2020). Pituitary apoplexy causing acute ischemic stroke: Which treatment should be given priority. Surg. Neurol. Int..

[212] Romano A., Ganau M., Zaed I., Scibilia A., Oretti G., Chibbaro S. (2020). Primary Endoscopic Management of Apoplexy in a Giant Pituitary Adenoma. World Neurosurg..

[213] Yang C., Han X., Du Y., Ma A.-Q. (2020). Takotsubo cardiomyopathy and pituitary apoplexy: A case report. BMC Cardiovasc. Disord..

[214] Alkhaibary A., Alsubaie N., Alharbi A., Alghanim N., Khairy S., Almuntashri M., Alwohaibi M., Alarifi A., Aloraidi A., Alkhani A. (2021). Oculomotor nerve palsy following coronary artery bypass graft surgery: Can pituitary apoplexy complicate the post-operative course of cardiac surgery?. J. Surg. Case Rep..

[215] Bhogal S., Patel N., Mawa K., Ramu V., Paul T. (2021). A Rare Case of Myxedema Coma Presenting as Bradycardia and Hypotension Secondary to Pituitary Apoplexy. Cureus.

[216] Elsehety M.A., Zeineddine H.A., Barreto A.D., Blackburn S.L. (2021). Failed endovascular therapy for acute internal carotid artery occlusion from pituitary apoplexy: Illustrative case. J. Neurosurgery: Case Lessons.

[217] Pokhrel B., Khanal S., Chapagain P., Sedain G. (2021). Pituitary Apoplexy Complicated by Cerebral Infarction: A Case Report. J. Nepal Med. Assoc..

[218] Steinberg J., Cohen J.E., Gomori J.M., Fraifeld S., Moscovici S., Rosenthal G., Shoshan Y., Itshayek E. (2013). Superficial siderosis of the central nervous system due to chronic hemorrhage from a giant invasive prolactinoma. J. Clin. Neurosci..

[219] Uemura M., Miyashita F., Shimomura R., Fujinami J., Toyoda K. (2013). Pituitary apoplexy during treatment with dabigatran. Neurol. Clin. Neurosci..

[220] Machado M.C., Gadelha P.S., Bronstein M.D., Fragoso M. (2013). Spontaneous remission of hypercortisolism presumed due to asymptomatic tumor apoplexy in ACTH-producing pituitary macroadenoma. Arq. Bras. Endocrinol. Metabol..

[221] Sun T., Liu L., Sunnassee A., Zhuo L., Zhu S. (2013). Sudden death in custody due to pituitary apoplexy during long restriction in a sitting position: A case report and review of the literature. J. Forensic Leg. Med..

[222] Kinoshita Y., Tominaga A., Usui S., Arita K., Sugiyama K., Kurisu K. (2014). Impact of subclinical haemorrhage on the pituitary gland in patients with pituitary adenomas. Clin. Endocrinol..

[223] Wan X.-Y., Chen J., Wang J.-W., Liu Y.-C., Shu K., Lei T. (2022). Overview of the 2022 WHO Classification of Pituitary Adenomas/Pituitary Neuroendocrine Tumors: Clinical Practices, Controversies, and Perspectives. Curr. Med. Sci..

[224] Mete O., Wenig B.M. (2022). Update from the 5th Edition of the World Health Organization Classification of Head and Neck Tumors: Overview of the 2022 WHO Classification of Head and Neck Neuroendocrine Neoplasms. Head Neck Pathol..

[225] WHO Classification of Tumours Editorial Board (2022). WHO Classification of Endocrine and Neuroendocrine Tumours.

[226] Asa S.L., Mete O., Perry A., Osamura R.Y. (2022). Overview of the 2022 WHO Classification of Pituitary Tumors. Endocr. Pathol..

